# An endoplasmic reticulum stress-responsive nanocomposite hydrogel for diabetic wound healing through a fibroblast-immune cell dual regulation hub

**DOI:** 10.1186/s12951-025-03732-0

**Published:** 2025-10-24

**Authors:** Shaoying Gao, Tao Chen, Chengliang Deng, Gang Liu, Zairong Wei

**Affiliations:** 1 Department of Burns and Plastic Surgery, Affiliated Hospital of Zunyi Medical University, Zunyi, 563000 China; 2 State Key Laboratory of Molecular Vaccinology and Molecular Diagnostics Center for Molecular Imaging and Translational Medicine, School of Public Health, Xiamen University, Xiamen, 361102 China

**Keywords:** Endoplasmic reticulum stress, Immune regulation, Regeneration, Diabetic wounds, Proliferation

## Abstract

Diabetic wounds exhibit excessive endoplasmic reticulum stress (ERS), which can lead to fibroblast dysfunction, abnormal natural killer (NK) cell activation, and imbalanced macrophage polarization. ERS exerts a bidirectional regulatory effect on wound fibroblasts and immune cells; simply inhibiting ERS impedes wound tissue regeneration. Existing hydrogels cannot precisely regulate ERS and synergistically repair multicell functional defects. Monocyte Chemoattractant Protein-1 (MCP-1), synthesized by fibroblasts in diabetic wounds, is a key molecular regulator of ERS and fibroblast function and a hub for fibroblast-immune cell interactions. This study constructed histidine–chitosan–fibroblast growth factor receptor (FGFR) agonist peptide 1 (FAP1)–pGPU6/GFP/Neo MCP-1–shRNA plasmid (HCFD) nanoparticles. The HCFD nanoparticles were loaded onto 3-carboxyphenylboronic acid (PBA)-modified methyl acrylate gelatin (GelMA) hydrogel (GP), forming the nanocomposite hydrogel Gel–PBA–HCFD (GPHCFD). The GPHCFD nanocomposite hydrogel utilizes FAP1 to target FGFR on wound fibroblasts. Under conditions of excessive ERS, GPHCFD nanocomposite hydrogels were triggered to break boric acid bonds and protonate histidine imidazole, thereby achieving the precise release of the plasmid, which could stably knock out the MCP-1 gene in fibroblasts. Notably, GPHCFD exhibited excellent ERS-responsive functionality, significantly reducing MCP-1 expression and alleviating excessive ERS in fibroblasts under in vitro thapsigargin (Tg)-induced high ERS conditions and high ERS environments in diabetic wounds. GPHCFD improved fibroblast proliferation, migration, and collagen secretion functions. Furthermore, GPHCFD inhibited inflammatory factors of NK cells, including TNF-α and IFN-γ, as well as the aberrant proliferation of CD45^+^CD3^−^NK1.1^+^ NK cells by reducing the MCP-1-mediated activation of the CCR2 receptor on NK cells and ERS. Additionally, CD45^+^CD3^−^NK1.1^+^CD11b^−^ NK cells were encouraged to differentiate into mature CD45^+^CD3^−^NK1.1^+^CD11b^+^ NK cells by GPHCFD. Additionally, the GPHCFD nanocomposite hydrogel reduced the MCP-1-mediated activation of the CCR2 receptor on macrophages, thereby decreasing excessive ERS in macrophages and driving their polarization from M1-type CD68^+^iNOS^+^ cells to M2-type CD68^+^CD206^+^ cells. This GPHCFD nanocomposite hydrogel integrates ERS-responsive nanoparticle release, fibroblast gene editing, and immune reprogramming, offering a new therapeutic modality for diabetic wounds incorporating tissue regeneration–immune homeostasis synergistic regulation.

**Graphical abstract:**

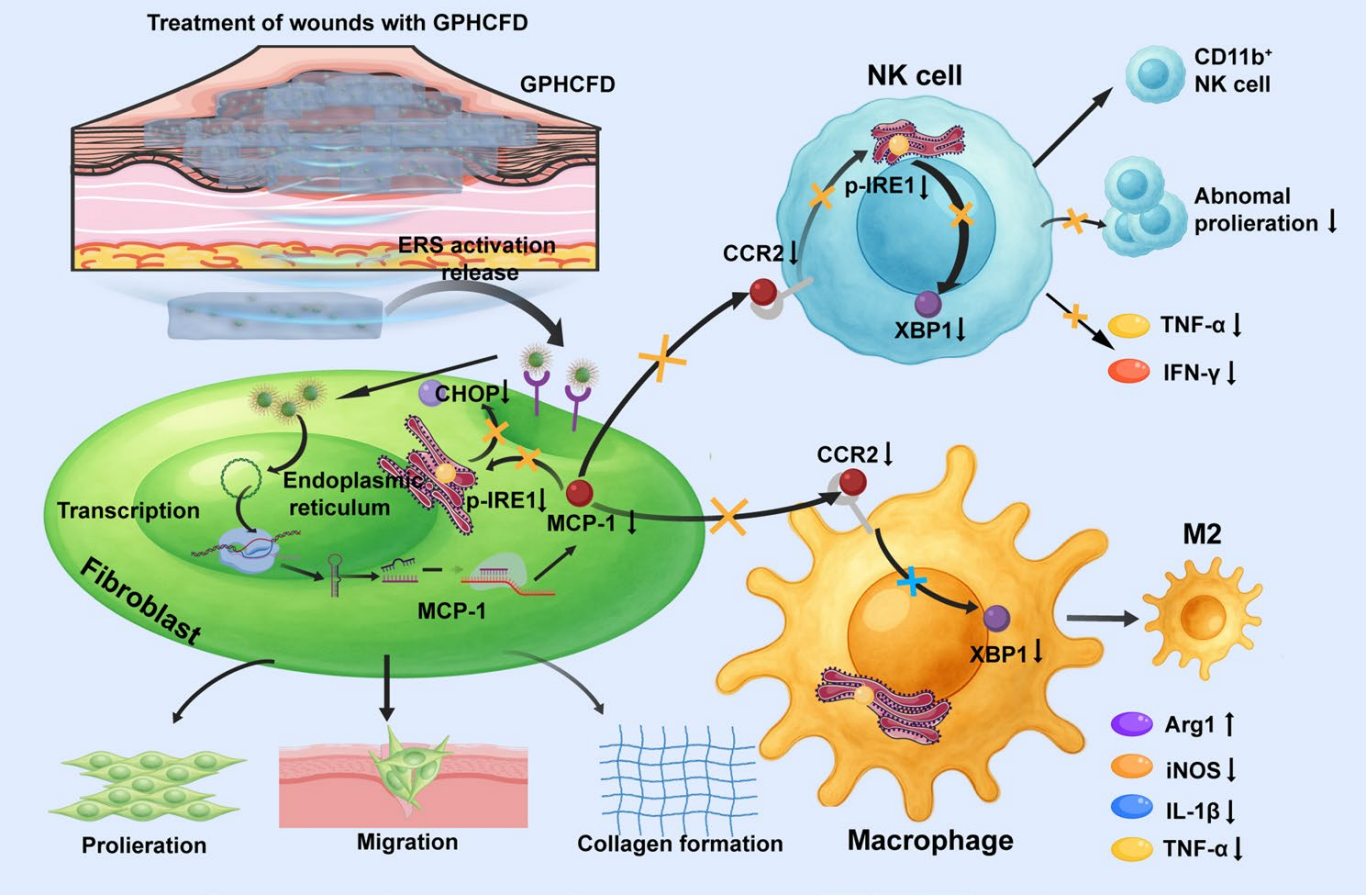

**Supplementary Information:**

The online version contains supplementary material available at 10.1186/s12951-025-03732-0.

## Introduction

Diabetic wounds that fail to heal represent a serious clinical complication [1]. The core pathophysiological mechanisms underlying diabetic wounds involve the dysfunction of key tissue repair cells and persistent local immune microenvironment disruption [2]. Endoplasmic reticulum stress (ERS) describes a condition of endoplasmic reticulum dysfunction caused by excessive formation of unfolded proteins in the endoplasmic reticulum [3]. The essence of ERS is protein homeostasis imbalance [4]. Moderate ERS activation activates protective pathways, while excessive ERS impairs the function of fibroblasts and immune cells through different mechanisms, delaying the recovery of diabetic injuries [5, 6]. Latest research has demonstrated that the high glucose levels, AGE accumulation, and chronic inflammation associated with diabetes can induce excessive ERS, which is a key mechanism impairing the repair of damaged tissue [7]. These important cells’ combined compromised function creates a vicious cycle: on the one hand, the structural and signaling support required for tissue regeneration is severely hindered, particularly due to fibroblast dysfunction [8]; on the other hand, the delicate immune regulatory balance between NK cell and macrophage dysfunction is profoundly disrupted, leading to unresolved inflammation and impaired repair signaling [9–11]. This dual impact of impaired tissue regeneration capacity and sustained disruption of the immune regulatory network ultimately results in the clinical challenge of chronic nonhealing wounds in diabetes.

Compared to plasmid-loaded nanoparticles such as liposomes, PLGA, or metal nanoparticles, chitosan nanoparticles not only exhibit excellent biocompatibility and degradability but also possess antibacterial and hemostatic capabilities [12, 13]. Simultaneously, chitosan synergistically enhances the therapeutic plasmids it carries, achieving dual functions of “self-therapeutic delivery” and “gene therapy” within the complex and challenging microenvironment of diabetic wounds [12]. Moreover, compared to common hydrogels based solely on gelatin, alginate, or polyethylene glycol, GelMA is particularly suited for diabetic wound treatment due to its exceptional biological activity and customizability [14]. Derived from gelatin, GelMA fully retains the RGD peptide sequence that promotes cell adhesion, migration, and proliferation [15]. Simultaneously, its physical properties—such as hardness, porosity, and degradation rate—can be precisely controlled [16]. Moreover, GelMA’s high functionalizability enables it to serve as a “multifunctional platform,” readily integrating various nanoparticles to synergistically address multiple challenges in diabetic wound healing, including infection, inflammation, and impaired angiogenesis [17]. Therefore, we selected chitosan and GelMA to prepare a nanocomposite hydrogel for wound treatment. Although existing nanocomposite hydrogels have made progress in the treatment of diabetic wounds [18], design flaws make it difficult to achieve therapeutic efficacy. These hydrogels lack ERS-responsive smart modules and cannot target drug delivery when ERS is overactivated to reduce ERS levels in fibroblasts. Furthermore, the absence of nanoparticles targeting fibroblasts increases the risk of off-target silencing, reducing gene therapy efficiency and potentially damaging surrounding cells. The inability to specifically regulate key genes in fibroblasts, for example, by knocking out MCP-1, limits their proliferative and collagen regeneration functions. Most materials rely on passive drug release mechanisms that do not readily adapt dynamically to changes in the wound microenvironment. The inability to dynamically intervene in the fibroblast-immune cell interaction axis pathway prevents the recognition and regulation of excessive ERS responses in immune cells such as NK cells and macrophages, thereby hindering further functional improvement. Consequently, developing strategies that intelligently sense and bidirectionally regulate the level of heat shock responses in immune cells has become an urgent need for enhancing immune cell function in diabetic wounds.

Previous studies have identified MCP-1, which is highly expressed in diabetic wound fibroblasts, as a key molecular regulator of ERS and fibroblast function and a hub for fibroblast-immune cell interactions [5, 19]. MCP-1/CCR2-mediated interactions between fibroblasts and immune cells impair macrophage and NK cell function [20]. In our prior studies, we demonstrated that local fibroblasts in diabetic wounds highly express FGFR and MCP-1; NK cells and macrophages highly express CCR2; and fibroblasts interact with NK cells and macrophages through the MCP-1/CCR2 pathway (Fig. S1A–F). Additionally, we found that the FAP1 (fibroblast activation protein 1) peptide is used for specific recognition of FGFR [21], which is highly expressed in wound fibroblasts [21]. Overexpression of the ERS triggers borate bond cleavage and histidine imidazole protonation [22–25].

**Scheme 1:**
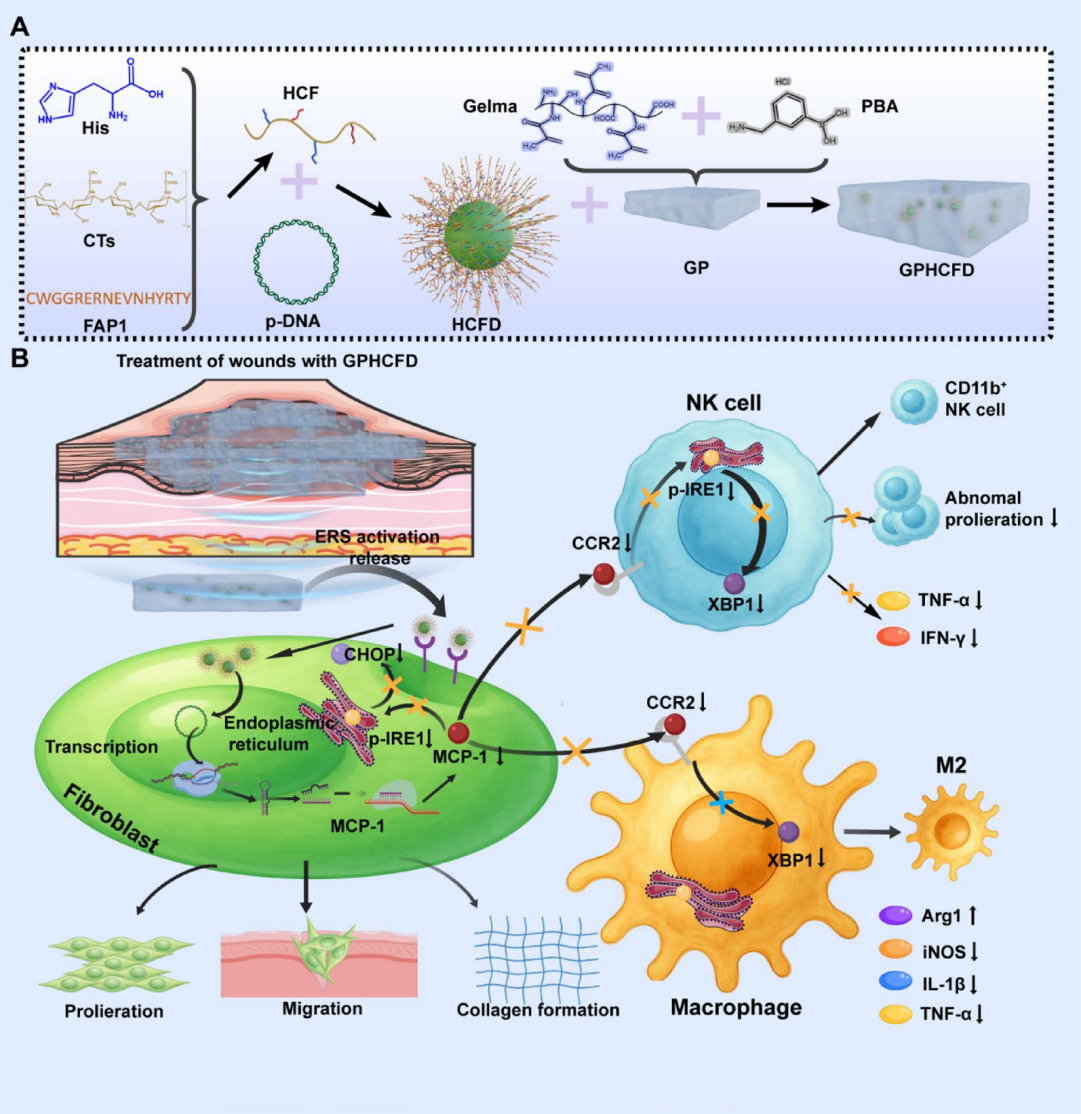
Schematic diagram illustrating the preparation of the GPHCFD hydrogel and its application to diabetic wounds. **(A)** Preparation of the GPHCFD nanocomposite hydrogel. **(B)** The GPHCFD nanocomposite hydrogel releases HCFD nanoparticles after excessive ERS activation. HCFD nanoparticles target fibroblasts, release pDNA plasmids, and target MCP-1 knockout, inhibiting ERS in fibroblasts by suppressing the ERS-specific signaling pathway p-IRE1/CHOP, thereby promoting fibroblast proliferation, migration, and collagen formation. This reduces the activation of the CCR2 of NK cells and the ERS-specific signaling pathway p-IRE1/XBP1 by MCP-1. It promotes the differentiation of NK cells into mature CD11b^+^ NK cells. This inhibits the abnormal proliferation of NK cells and the expression of proinflammatory proteins IFN-γ and TNF-α. By reducing the effect of MCP-1 on CCR2 in macrophages, inhibiting the ERS-specific proteins IRE1 and XBP1 reduces ERS in these cells, promoting their conversion from the M1 to the M2 phenotype

Based on this, we constructed the pGPU6/GFP/Neo MCP-1-shRNA plasmid (pDNA) to stably knock out the MCP-1 gene in fibroblasts and developed histidine (His)–chitosan (CTs)–FAP1–pDNA (HCFD) nanoparticles. HCFD nanoparticles were loaded onto 3-carboxyphenylboronic acid (PBA)-modified methyl acrylate gelatin (GelMA) hydrogel (GP), forming the nanocomposite hydrogel Gel–PBA–HCFD (GPHCFD) (Scheme 1A). Excessive ERS triggered the release of pDNA from GPHCFD. Notably, GPHCFD reduced MCP-1 expression intelligently, inhibited fibroblast ERS, and thereby restored fibroblast proliferation, migration, and collagen secretion functions. Furthermore, by dynamically interfering with MCP-1/CCR2 signaling in the fibroblast–immune cell interaction axis, GPHCFD decreased the ERS levels of macrophages and NK cells, prevented NK cells from secreting inflammatory factors, stopped CD45^+^CD3^−^NK1.1^+^ NK cells from proliferating abnormally, encouraged the maturation of CD11b^−^CD45^+^CD3^−^NK1.1^+^ NK cells, and drove M1 to M2 macrophage conversion (Scheme 1B). In summary, this study provides a new therapeutic model for diabetic wounds involving tissue regeneration–immune homeostasis synergistic regulation through the combination of three technologies: ERS-responsive nanoparticle controlled release, fibroblast-targeted gene editing, and immune microenvironment reprogramming.

## Results

### Preparation and characterization of HCFD nanoparticles

**Fig. 1:**
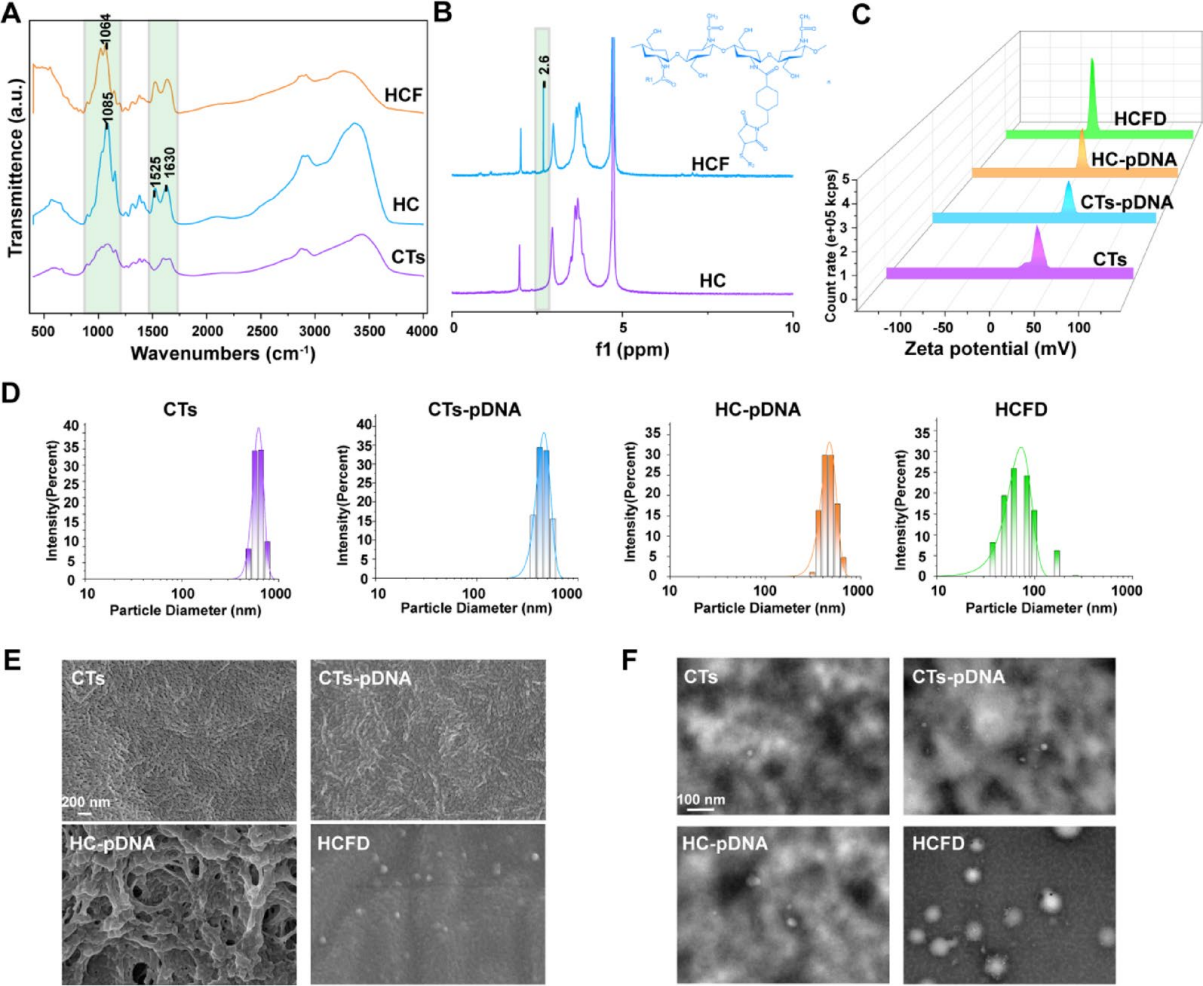
Preparation and characterization of HCFD nanoparticles. **(A)** Infrared spectroscopy analysis of CTs, HC, and HCF. **(B)** Nuclear magnetic resonance hydrogen spectroscopy analysis of HC and HCF. **(C)** Zeta potential measurement of CTs, CTs–pDNA, HC–pDNA, and HCFD. **(D)** Particle size distribution of CTs, CTs–pDNA, HC–pDNA, and HCFD. **(E)** SEM analysis of CTs, C–pDNA, HC–pDNA, and HCFD. **(F)** TEM analysis of CTs, C–pDNA, HC–pDNA, and HCFD

To confirm that FAP1 and His were successfully attached to CTs, we performed infrared spectroscopy on CTs, HC (His-CTs), and HCF (His-CTs-FAP1 peptide). Compared to CTs, HC exhibits an enhanced absorption peak at 1085 cm^−1^ in its infrared spectrum. This peak may be associated with vibrations of glycosyl or His residues, indicating the formation of new bonds [26–28]. The characteristic peak shift at 1525 cm^−1^ and the enhanced absorption peak at 1630 cm^−1^ correspond to the amide I band (C = O stretching vibration), which typically indicates the formation or strengthening of amide bonds. This phenomenon is commonly observed during peptide-polymer conjugation and serves as a chemical signature for the binding of proteins or peptides to carriers such as CTs. The observed enhancement may originate from the covalent linkage between His peptides and CTs (Fig. 1A) [29]. Compared to HC, the infrared spectrum of HCF exhibits reduced intensity at the characteristic peak at 1064 cm⁻¹. This reduction may be attributed to the formation of an intermolecular hydrogen bond between the guanidino group (-NH-C(= NH)NH₂) of the arginine residue in the FAP1 peptide and the phenolic hydroxyl group (-OH) of tyrosine (Fig. 1A) [30]. We further validated this effect using nuclear magnetic resonance (NMR) spectroscopy. The results revealed that compared to the HC group, the NMR spectra of the HCF group exhibited a new peak at 2.6 ppm, likely originating from the introduction of methylene or methyl groups from the FAP1 peptide (Fig. 1B) [31]. These findings suggested that FAP1 and His were successfully conjugated to CTs.

We constructed the plasmid pGPU6/GFP/Neo MCP-1-shRNA to stably knock out MCP-1. To verify whether pDNA was successfully loaded onto HCF nanoparticles, we measured the zeta potential of CTs, CTs-pDNA, HC-pDNA, and HCF-pDNA (HCFD). Unlike CTs, CTs-pDNA, HC-pDNA, and HCFD exhibited a negative potential (Fig. 1C), consistent with the Phase Image (Fig. S2). We further measured the particle size changes of CTs, HC, and HCF, and found that that of HCFD nanoparticles primarily ranged from 50 to 100 nm (Fig. 1D). The morphological alterations of pDNA following binding to CTs, HC, and HCF were investigated using scanning electron microscopy (SEM) and transmission electron microscopy (TEM). In contrast to CTs, CTs–pDNA, and HC–pDNA, HCFD produced spherical particles that were mainly between 60 and 90 nm in diameter, which is in line with the particle size range that DLS detected (Fig. 1E, F). The above results indicated that compared to CTs and HC, HCF is more readily capable of forming spherical carriers with pDNA. The main particle size of these spherical carriers is less than 100 nanometers, which helps them enter cells more effectively and perform their functions.

### Synthesis and physical and chemical characterization of the GPHCFD hydrogel

**Fig. 2:**
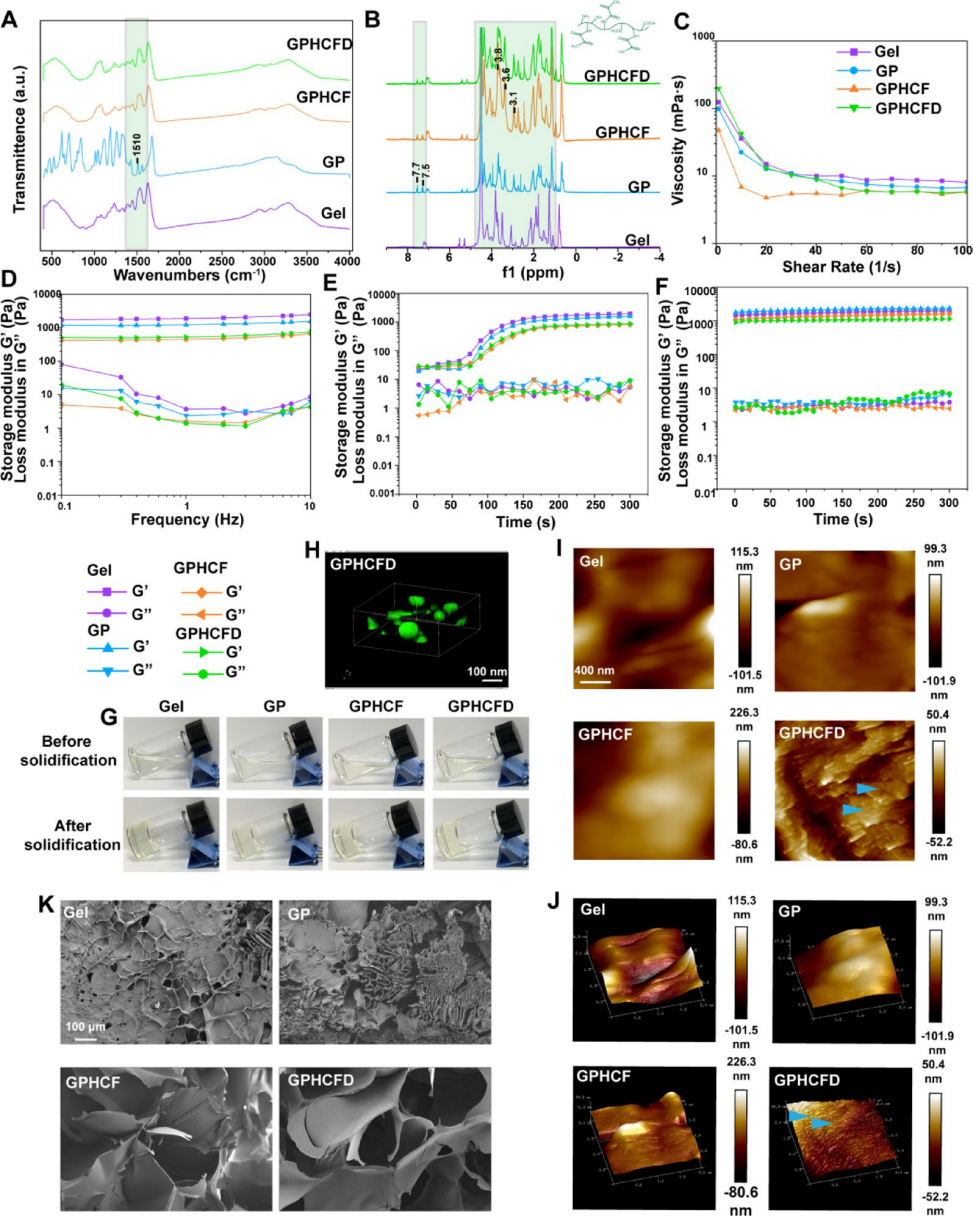
Synthesis and physicochemical characterization of the GPHCFD hydrogel. **(A)** Infrared spectroscopy analysis of the Gel, GP, GPHCF, and GPHCFD hydrogels. **(B)** Nuclear magnetic resonance hydrogen spectroscopy analysis of the Gel, GP, GPHCF, and GPHCFD hydrogels. **(C)** Viscosity measurements of the Gel, GP, GPHCF, and GPHCFD hydrogels. **(D)** Frequency sweep analysis of the Gel, GP, GPHCF, and GPHCFD hydrogels. **(E)** Photocuring time analysis of the Gel, GP, GPHCF, and GPHCFD hydrogels. **(F)** Self-healing analysis of the Gel, GP, GPHCF, and GPHCFD hydrogels. **(G)** Typical images of the Gel, GP, GPHCF, and GPHCFD hydrogels before and after curing. **(H)** Fluorescence confocal microscopy of nanoparticles in GPHCFD hydrogel. **(I**,** J)** AFM analysis of the Gel, GP, GPHCF, and GPHCFD liquid hydrogels. **(K)** SEM images of the Gel, GP, GPHCF, and GPHCFD hydrogels

We performed infrared spectroscopy on Gelma (Gel), GP, GPHCF, and GPHCFD to verify the successful formation of the GPHCFD hydrogel. In the mid-infrared region, the characteristic peak at 1510 cm⁻¹ corresponded to the vibration of the benzene ring skeleton, indicating the successful incorporation of benzene-containing components into the hydrogel [32] (Fig. 2A). The infrared spectra of GPHCF and GPHCFD showed no changes in comparison with that of GP, which was attributable to the excessive number of groups attached to GP in GPHCF and GPHCFD (Fig. 2A) [33].

In contrast to GelMA, GP showed additional peaks at 7.7 ppm and 7.5 ppm, which correspond to the ortho and para protons of the PBA benzene ring, respectively, according to a nuclear magnetic resonance hydrogen spectroscopy study [34]. The shift at 7.5 ppm indicates enhanced polarity of the B-O bond, leading to a conjugation effect, which is consistent with the expected formation of a borate ester bond (B-OR) with PBA (Fig. 2B). This reflects the formation process of dynamic covalent bonds. At the same time, the enhanced peaks at 3.1, 3.6, and 3.8 ppm in the H_3_-H_6_ proton region of the CTs sugar ring may indicate intermolecular interactions, such as hydrophobic effects, consistent with the mechanism of chitosan binding to FAP1 (Fig. 2B) [34]. Furthermore, the high-molecular-weight matrix background obscures the pDNA signal; therefore, there is no discernible difference between the GPHCFD and GPHCF spectra [35]. The aforementioned findings suggested the successful synthesis of GPHCFD.

We performed rheological characterization of Gel, GP, GPHCF, and GPHCFD hydrogels. Shear rate scanning tests showed that within the γ̇ = 1–10 s^−1^ range (corresponding to injection rates of 0.1-1 mL/min), GPHCF and GPHCFD exhibited significant shear thinning behavior, with viscosity (η) maintained at 7–20 mPa·s (Fig. 2C), confirming their injectability [36]. The development of a stable gel network was demonstrated by oscillation time scans. It showed that GPHCFD’s storage modulus (G’) remained higher than its loss modulus (G’’) (Fig. 2D). The curing time of GPHCFD hydrogel, influenced by the added CTs, ranged from 50 to 80 seconds (Fig. 2E, G). Frequency scanning revealed that the G’ value of GPHCFD hydrogel is below 1000 Pa (Fig. 2F), approaching the mechanical properties of soft tissues (e.g., adipose tissue ~ 300 Pa), which is advantageous for subsequent biomedical applications [37].

Fluorescence confocal microscopy was used to generate 3D images of fluorescently labeled HCFD nanoparticles in GPHCFD hydrogels, demonstrating that HCFD nanoparticles had successfully attached to the GPHCFD hydrogels (Fig. 2H). Atomic force microscopy (AFM) analysis of 2D and 3D images of the Gel, GP, GPHCF, and GPHCFD hydrogels revealed that only GPHCFD exhibited a distinct granular texture. GPHCFD contained a large number of HCFD nanoparticles (Fig. 2I, J), with particle sizes ranging from 50 to 90 nm (Fig. S3). SEM analysis was used to examine the morphology of the Gel, GP, GPHCF, and GPHCFD hydrogels. Adding a pore-forming agent during GP synthesis and linking HCF and HCFD resulted in GPHCF and GPHCFD hydrogels exhibiting larger pores (Fig. 2K). The synthesized GPHCFD hydrogel facilitated the effective release of HCFD nanoparticles. These results suggested the successful assembly of GPHCFD hydrogels (loaded HCFD nanoparticles). These results also indicated that the GPHCFD hydrogels were suitable for subsequent nanoparticle delivery and cell experiments.

### Biological function testing of ERS-responsive GPHCFD hydrogels

**Fig. 3:**
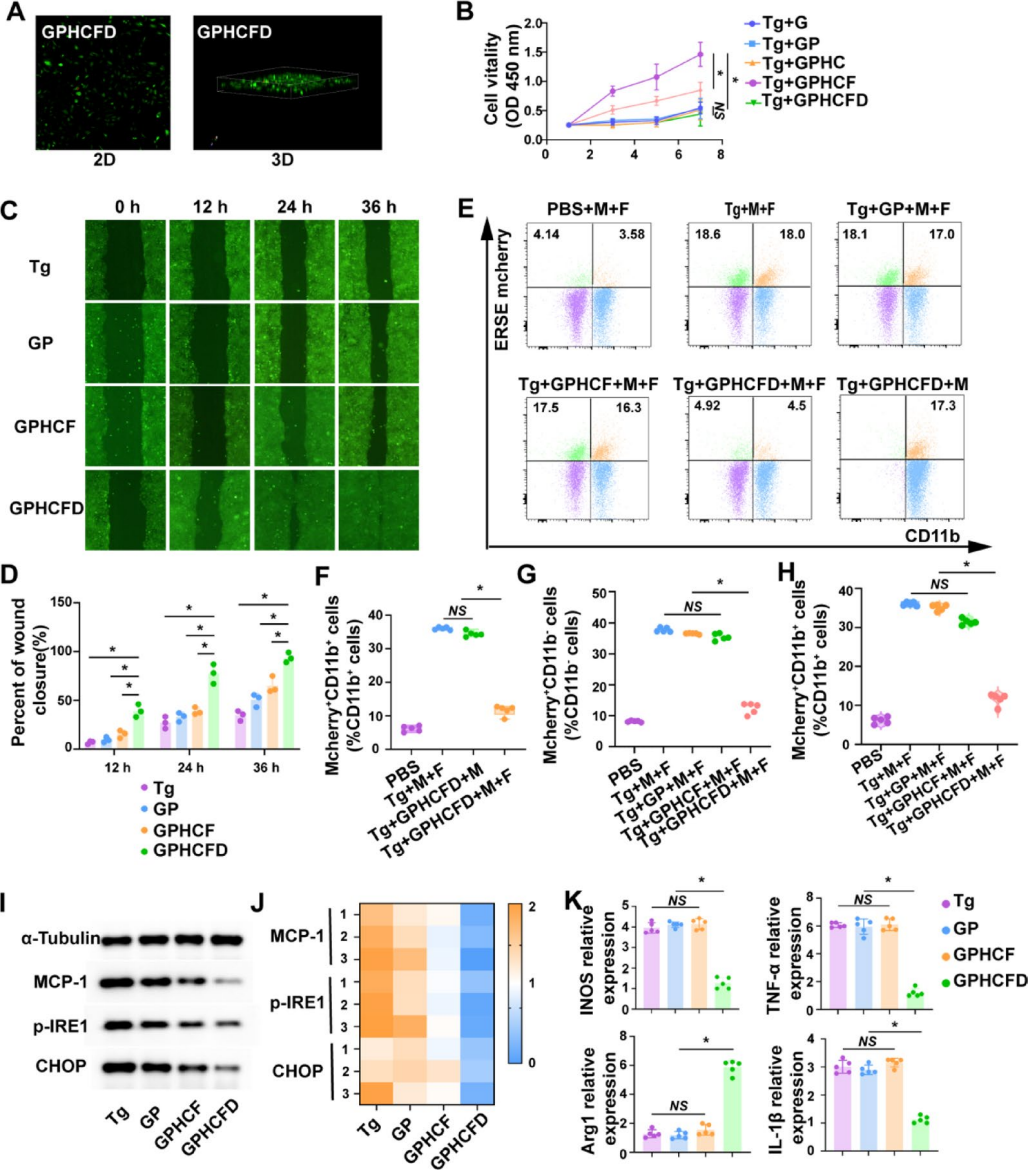
Biological function testing of ERS-responsive GPHCFD hydrogels. **(A)** Fluorescence confocal detection of fibroblast proliferation in the GPHCFD hydrogels. Red: dead fibroblast; green: live fibroblast. **(B)** CCK8 detection of fibroblast proliferation in the Gel GP, GPHCF, and GPHCFD hydrogels induced by Tg on days 1, 3, 5, and 7, in that order. *n* = 3 per group. **(C**,** D) **Tg-induced fibroblasts produce excessive ERS; scratch assay of fibroblast migration in the no hydrogel, GP, GPHCF, and GPHCFD hydrogel-treated teams. *n* = 3 per team. **(E)** Flow cytometry assessment of the PBS group without Tg induction, the Tg-induced fibroblast (F) and macrophage (M) group, the GP hydrogel-treated Tg-induced fibroblast and macrophage group, the GPHCF hydrogel-treated Tg-induced fibroblast and macrophage group, the GPHCFD hydrogel-treated Tg-induced fibroblast and macrophage group, and the GPHCFD hydrogel-treated Tg-induced macrophage group. **(F)** The proportion of mcherry^+^CD11b^+^ cells within CD11b^+^ cells is statistically analyzed. *n* = 5 per team. *NS*: non-significant, *P < 0.05. **(G)** The proportion of mcherry^+^CD11b^−^ cells within CD11b^−^ cells is statistically analyzed. **(H)** The proportion of mcherry^+^CD11b^+^ cells within CD11b^+^ cells is statistically analyzed. *n* = 5 per group. **(I)** Tg-induced four groups without hydrogels; GP, GPHCF, and GPHCFD hydrogel-treated fibroblasts and macrophages underwent ERS. Western blotting was used to assess the expression levels of MCP-1, p-IRE1, and CHOP in fibroblasts from the four groups. **(J)** The statistical analysis was performed via a heatmap. *n* = 3 per group. **(K)** Fluorescent quantitative PCR was used to measure the expression levels of TNF-α, iNOS, IL-1β, and Arg1 in macrophages from the four groups. *n* = 5 per team. *NS*: non-significant, *P < 0.05. The data is displayed as mean ± SD.

Fluorescence confocal imaging of fibroblasts cultured on GPHCFD hydrogels (2D/3D) showed multidirectional growth (green) and minimal cell death (red) (Fig. 3A), indicating good biocompatibility. The GPHCD hydrogel exhibited the strongest promotion of fibroblast cell proliferation when fibroblast cells were induced to undergo ERS in vitro using Tg. Only GPHCFD and GPHCF hydrogels demonstrated a significant promotional effect on fibroblast proliferation. Therefore, we selected GPHCD and GPHCF as the experimental groups and GP as the control group for the subsequent experiments (Fig. 3B). These results suggest that excessive ERS can activate GPHCFD hydrogel release plasmid, knock down the MCP-1 gene in fibroblasts, and inhibit the expression of MCP-1. Induce the ERS in fibroblasts using Tg concentrations of 0 mM, 10 mM, 50 mM, and 100 mM. Using flow cytometry, assess the fluorescence intensity of cells transfected with a GFP-labeled plasmid. The GFP fluorescence intensity was significantly increased in the 50 mM and 100 mM Tg induction groups, with the highest GFP fluorescence intensity observed in the 100 mM Tg induction group. This indicated that as ERS increased in fibroblasts, plasmid transfection efficiency was significantly enhanced (Fig. S4). Among the four groups of Tg-induced fibroblasts that underwent ERS–the non-hydrogel group, GP group, GPHCF group, and GPHCFD hydrogel group–the GPHCFD hydrogel group had the fastest migration speed of fibroblasts (Fig. 3C, D).

Fibroblast (F) and macrophage (M) cell lines expressing ESRE mCherry were established. When excessive ERS occurs, both fibroblasts and macrophages show positive expression of ESRE mCherry. In the coculture system, CD11b^+^ cells represented macrophages, and CD11b^−^ cells represented fibroblasts. The following groups were analyzed: PBS without Tg induction, Tg-induced fibroblasts and macrophages, Tg-induced macrophages, GP hydrogel-treated Tg-induced fibroblasts and macrophages, GPHCF hydrogel-treated Tg-induced fibroblasts and macrophages, and GPHCFD hydrogel-treated Tg-induced fibroblasts and macrophages. The proportion of ESRE mCherry^+^CD11b^+^ cells was significantly higher in Tg-induced fibroblasts and macrophages and in Tg-induced macrophages, whereas the proportion of ERSE mCherry^+^CD11b^+^ cells was very low in the non-Tg-induced PBS group and in hydrogel-treated Tg-induced fibroblasts and macrophages. This finding indicated that the GPHCFD hydrogel was ERS-responsive and could only exert its ERS-inhibitory function in the presence of fibroblasts. The GPHCFD hydrogel targeted fibroblasts and inhibited ERS in both fibroblasts and macrophages (Fig. 3E, F). The proportion of ERSE mcherry^+^CD11b^−^ cells was high in the Tg-induced fibroblast and macrophage groups, the GP hydrogel-treated Tg-induced fibroblast and macrophage groups, and the GPHCF hydrogel-treated Tg-induced fibroblast and macrophage groups. In contrast, the proportion of ERSE mcherry^+^CD11b^−^ cells was low in the non-Tg-induced PBS group and the GPHCDFD hydrogel-treated Tg-induced fibroblast and macrophage group (Fig. 3E, G). While the non-Tg-induced PBS group and the GPHCFD hydrogel-treated Tg-induced fibroblast and macrophage groups showed a low proportion of mcherry^+^CD11b^+^ cells, the Tg-induced fibroblast and macrophage group, the GP hydrogel-treated Tg-induced fibroblast and macrophage group, and the GPHCF hydrogel-treated Tg-induced fibroblast and macrophage group showed a high proportion of ERSE mcherry^+^CD11b^+^cells (Fig. 3E, H), suggesting that the ERS-responsive GPHCFD can simultaneously inhibit ERS in fibroblasts and macrophages. Tg-induced fibroblasts and macrophages underwent ERS. A coculture system was established with two cell types divided into the no hydrogel, GP, GPHCF, and GPHCFD hydrogel-treated groups. Western blotting analysis of fibroblasts in the coculture system revealed that the GPHCFD group exhibited the lowest expression levels of MCP-1 and the ERS-specific molecules p-IRE1 and CHOP among the four groups, confirming that the GPHCFD hydrogel can inhibit MCP-1 expression in fibroblasts and reduce fibroblast ERS levels (Fig. 3I, J, S5). The GPHCFD group showed the greatest abundance of the M2-type characteristic molecule Arg1 and the lowest expression of the M1-type characteristic molecules TNF-α, iNOS, and IL-1β, according to fluorescent quantitative PCR analysis of macrophages in the coculture system. This suggested that the GPHCFD hydrogel can prevent the promotion of M1 to M2 macrophage transformation (Fig. 3K). The above results demonstrated that the GPHCFD hydrogel is an ERS-responsive hydrogel. By suppressing MCP-1 expression, the GPHCFD hydrogel could decrease excessive ERS in fibroblasts and encourage fibroblast migration and proliferation. It could also decrease ERS in macrophages and encourage the polarization of M1-type macrophages toward the M2 phenotype.

### The GPHCFD hydrogel promoted diabetic wound healing

**Fig. 4:**
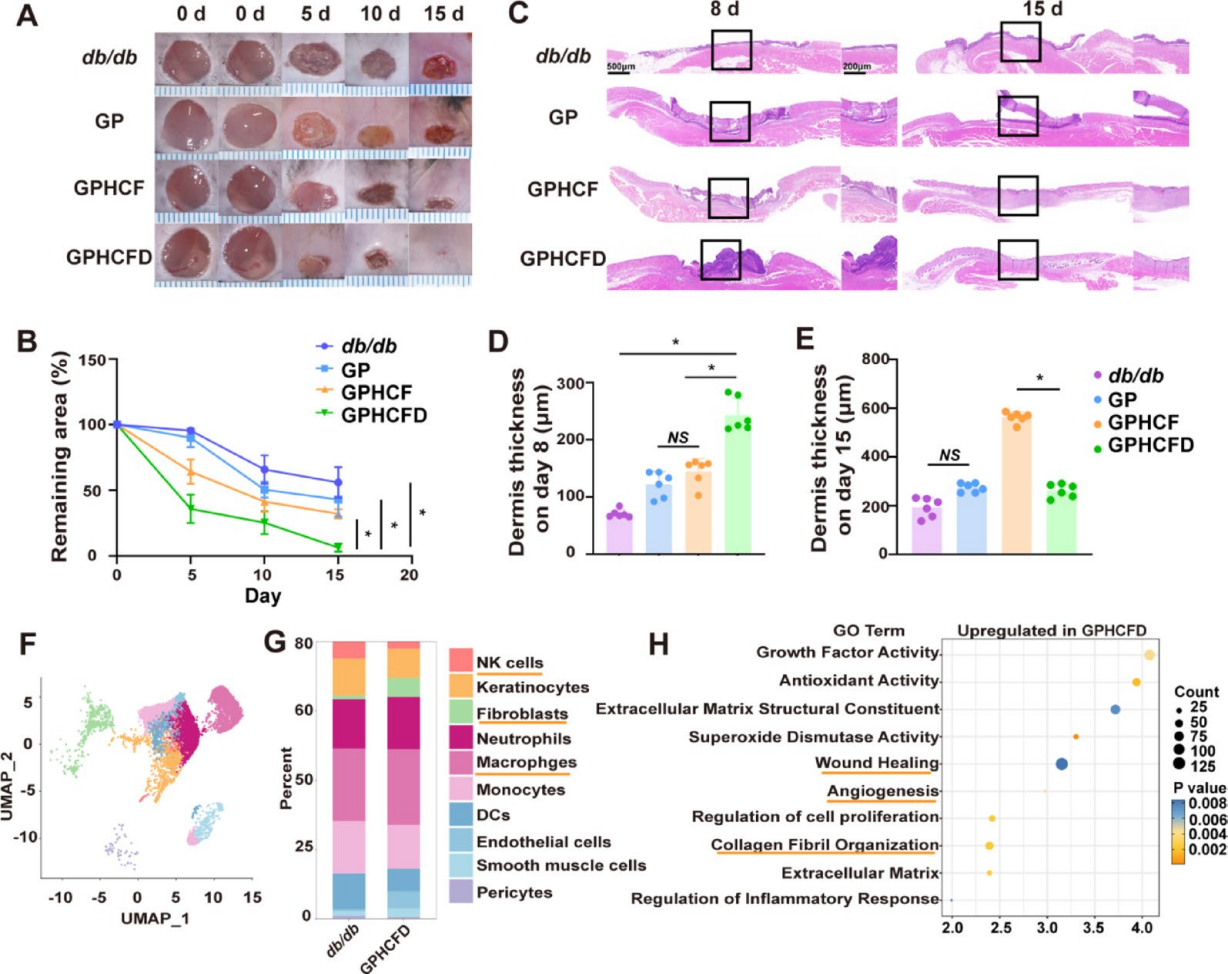
The GPHCFD hydrogel facilitated diabetic wound healing. **(A)** Typical images of diabetic wounds treated with no hydrogel, GP, GPHCF, and GPHCFD hydrogel at days 5, 10, and 15. *n* = 6 per group. **(B)** Statistical analysis of the wound areas. **(C)** H&E stained images showing wound healing in the no hydrogel, GP, GPHCF, and GPHCFD hydrogel treatment groups for diabetic wounds. Scale bar, 1000 μm and 200 μm. **(D**,** E)** Statistical analysis of dermal thickness in the four groups at days 8 and 15. *n* = 6 in each group. **(F)** UMAP plot analysis of 10 cell clusters in the GPHCFD hydrogel-treated diabetic wound group. **(G)** Bar chart analysis of the proportion of 10 cell clusters in the non-hydrogel-treated diabetic wound group and the GPHCFD hydrogel-treated diabetic wound group. **(H)** GO enrichment analysis of wound healing-related gene sets in the GPHCFD hydrogel-treated diabetic wound group. *n* = 3 per team. The data is displayed as mean ± SD.

We further investigated the therapeutic effects of the GPHCFD hydrogel on diabetic wounds. Three hydrogels, namely, GP, GPHCF, and GPHCFD, were used to treat diabetic wounds, and the wound areas of the untreated, GP, GPHCF, and GPHCFD groups were assessed. GPHCFD hydrogel and GPHCF hydrogel significantly promoted wound contraction in comparison with that observed in the untreated group, with the GPHCFD hydrogel showing the best therapeutic effect (Fig. 4A, B). HE staining was used to assess changes in the dermis of the untreated, GP, GPHCF, and GPHCFD hydrogel groups. The GPHCFD and GPHCF hydrogels promoted dermal formation, with the most significant dermal thickening observed in the GPHCFD hydrogel group on day 8 (Fig. 4C, D). By day 15, the dermis thickness in the GPHCFD hydrogel group had already begun to reduce (Fig. 4C, E), indicating that the dermis in the GPHCFD hydrogel group matured earliest. Additionally, on day 15, the GPHCFD hydrogel group exhibited more dermal hair follicles than the other three groups (Fig. 4C).

To explore the mechanism of action of the GPHCFD hydrogel on wounds, we performed single-cell sequencing on diabetic wound tissue without treatment on day 3 and diabetic wound tissue treated with GPHCFD hydrogel. Wound cells were classified into 10 cell groups: fibroblasts, macrophages, neutrophils, NK cells, keratinocytes, monocytes, endothelial cells, pericytes, DCs, and smooth muscle cells (Fig. 4F). The percentage of fibroblasts was much higher in GPHCFD hydrogel-treated diabetic wound tissue than in untreated diabetic wound tissue, but the percentage of NK cells was significantly lower (Fig. 4G). GO enrichment analysis related to wound healing showed that gene sets related to wound healing, collagen fibril organization, and angiogenesis were significantly enriched in the GPHCFD hydrogel treatment group (Fig. 4H). The results indicated that GPHCFD hydrogel promoted the healing of diabetic wounds, potentially by enhancing fibroblast proliferation and suppressing abnormal natural killer cell proliferation.

### The GPHCFD hydrogel inhibited excessive ERS in fibroblasts by suppressing MCP-1 expression, thereby promoting collagen formation

**Fig. 5:**
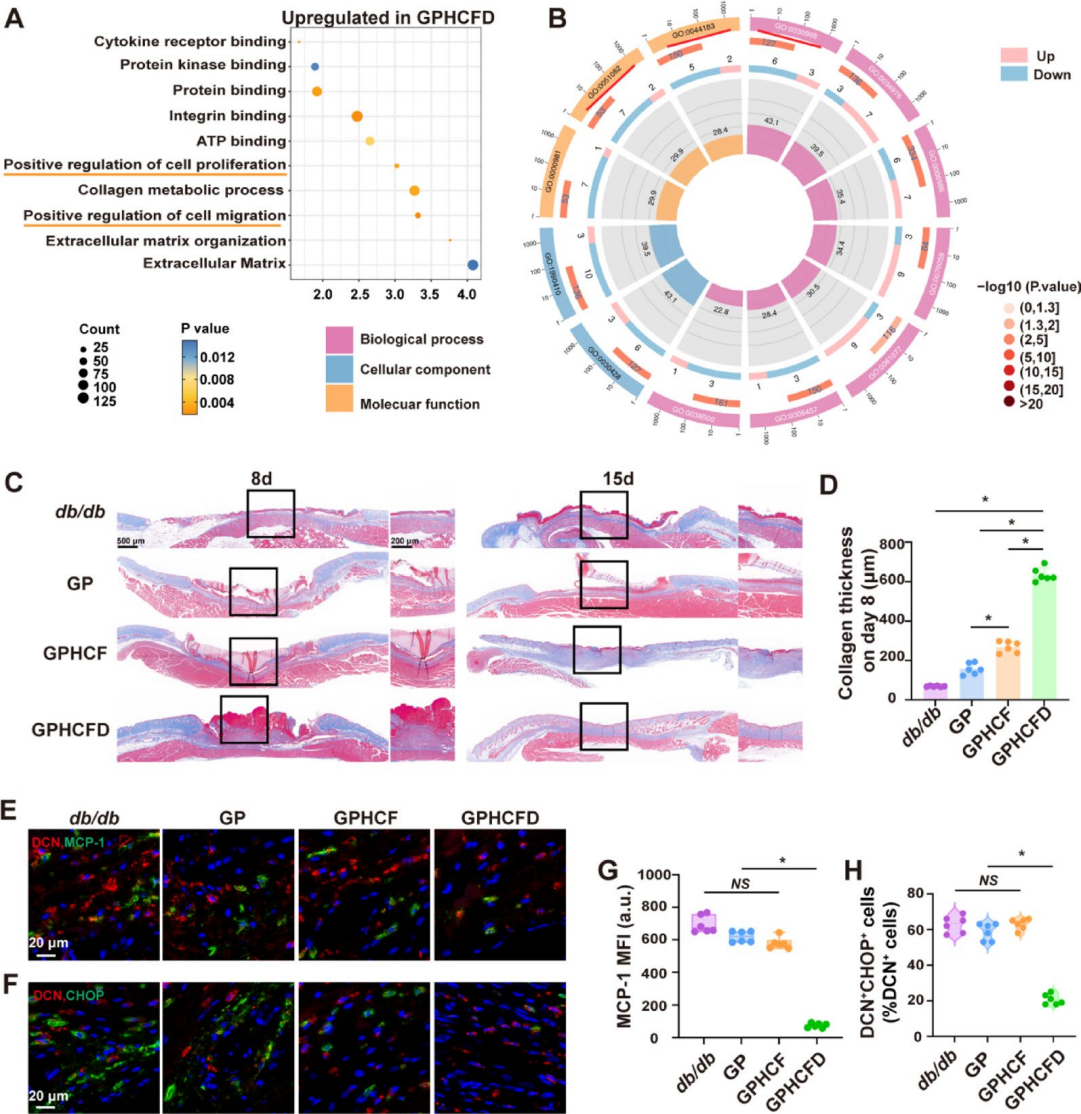
The GPHCFD hydrogel inhibits excessive ERS in diabetic wound fibroblasts by suppressing MCP-1 expression and promotes collagen formation. **(A)** GO enrichment analysis bubble chart analysis of wound healing-related functions in diabetic wound fibroblasts treated with the GPHCFD hydrogel. *n* = 3 per group. **(B)** GO enrichment circle plot analysis of ERS-related functions in local fibroblasts from diabetic wound tissue treated with the GPHCFD hydrogel. *n* = 3 per group. **(C)** Masson’s trichrome to detect collagen formation in four groups of diabetic wounds: no hydrogel treatment and GP, GPHCF, and GPHCFD hydrogel treatment on days 8 and 15. 500 μm and 200 μm Scale bar. **(D)** Statistical evaluation of collagen thickness on day 8. *n* = 6 per team. **(E)** Immunofluorescence detection of MCP-1 expression levels in diabetic wounds treated with the GPHCFD hydrogel (Red: DCN; Blue: nuclei; Green: MCP-1). Scale bar, 20 μm. **(F)** Immunofluorescence detection of DCN^+^CHOP^+^ cells in the local area of diabetic wounds treated with no hydrogel and GP, GPHCF, and GPHCFD hydrogels. Scale bar, 20 μm. **(G)** Statistical evaluation of MCP-1’s mean fluorescence intensity. *n* = 6 in each group. **(H)** The percentage of DCN^+^CHOP^+^ cells among DCN^+^ cells is statistically analyzed in the wound area. *n* = 6 per team. *NS*: non-significant, *P < 0.05. The data is displayed as mean ± SD.

We analyzed the functional roles of local fibroblasts in diabetic wound tissue treated with the GPHCFD hydrogels using single-cell sequencing data. GO enrichment analysis showed that gene sets related to positive regulation of cell proliferation and migration were significantly enriched in fibroblasts treated with the GPHCFD hydrogels (Fig. 5A). GO enrichment circle diagram analysis showed that in diabetic wound tissue, local fibroblasts treated with the GPHCFD hydrogels exhibited significant downregulation of genes related to ERS functions, including unfolded protein binding (GO:0051082), endoplasmic reticulum unfolded protein response (GO:0030968), and protein folding chaperone (GO:0044183) (Fig. 5B). These findings confirmed that the GPHCFD hydrogel might inhibit excessive ERS in the local fibroblasts of diabetic wounds, thereby promoting fibroblast proliferation and migration.

We further investigated the effect of the GPHCFD hydrogel on fibroblast collagen secretion. Masson’s trichrome was used to assess collagen formation on the diabetic wound surfaces of the four groups (no hydrogel treatment, GP, GPHCF, and GPHCFD hydrogel treatment) on days 8 and 15, and collagen thickness was statistically analyzed. In comparison to the collagen layers in the other three groups, the GPHCFD hydrogel treatment group’s layer was the thickest on day 8 (Fig. 5C, D). By day 15, because collagen in the GPHCFD hydrogel-treated group had begun to mature, the collagen layer in the GPHCF hydrogel-treated group was the thickest (Fig. 5C, S6). This finding suggested that the GPHCFD hydrogel significantly promoted collagen formation and maturation in fibroblasts. DCN is a characteristic marker of fibroblasts. We used immunofluorescence to assess the expression of MCP-1 and the characteristic ERS protein CHOP in diabetic wounds on day 3 in the untreated, GP, GPHCF, and GPHCFD hydrogel groups. In contrast to the remaining three groups, the GPHCFD group exhibited the lowest local MCP-1 expression level and the lowest proportion of DCN^+^CHOP^+^ cells in diabetic wounds (Fig. 5E, G). Western blot analysis was performed to detect MCP-1 expression levels at the wound sites across all groups. We similarly found that the GPHCFD hydrogel group exhibited the lowest MCP-1 expression levels compared to the other three groups (Fig. S7). This finding indicated that the GPHCFD hydrogel could reduce the expression level of MCP-1 and inhibit excessive ERS in fibroblasts at the wound site. The above results indicated that GPHCFD hydrogel promoted tissue regeneration in wounds from diabetes by preventing the expression of MCP-1 in fibroblasts, suppressing ERS in fibroblasts, and promoting collagen formation.

### The GPHCFD hydrogel inhibited excessive ERS in NK cells, thereby suppressing abnormal proliferation and inflammatory factor secretion in NK cells and promoting NK cell maturation

**Fig. 6:**
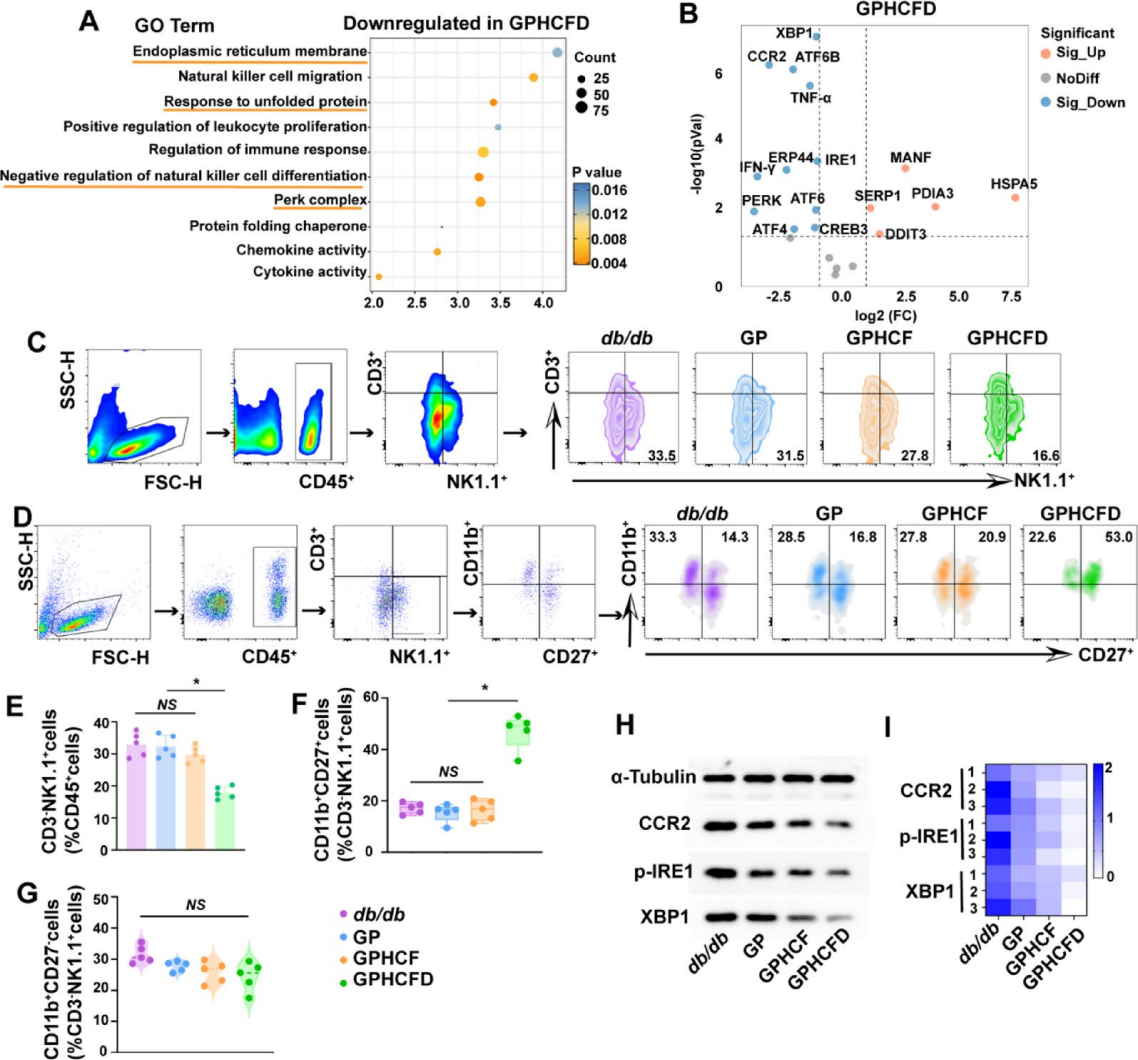
The GPHCFD hydrogel inhibits abnormal proliferation and inflammatory factor secretion of NK cells by suppressing excessive ERS, thereby promoting NK cell maturation. **(A)** GO enrichment analysis bubble chart analysis of ERS-related functions and NK cell differentiation functions in local NK cells of diabetic wound tissue treated with the GPHCFD hydrogel. *n* = 3 per group. **(B)** Volcano plot analysis of ERS-related gene expression and inflammatory factor expression in local NK cells of diabetic wound tissue treated with the GPHCFD hydrogel. *n* = 3 per team. **(C)** Flow cytometry was used to detect CD45^+^CD3^−^NK1.1^+^ NK cells from diabetic wounds in the untreated, GP, GPHCF, and GPHCFD groups. **(D)** Flow cytometry was used to detect CD11b^+^CD27^+^ NK cells and CD11b^+^CD27^−^ NK cells in diabetic wounds from the untreated group, GP group, GPHCF group, and GPHCFD group, respectively. **(E)** The percentage of CD45^+^CD3^−^NK1.1^+^ cells within CD45^+^ cells is statistically analyzed. *n* = 5 in each group. **(F)** Statistical analysis of the percentage of CD11b^+^CD27^+^ NK cells among CD3^−^NK1.1^+^ cells. *n* = 5 per group. **(G)** The percentage of CD11b^+^CD27^−^ NK cells among CD3^−^NK1.1^+^ cells. *n* = 5 per group. **(H)** Flow cytometry-sorted NK cells from diabetic wounds in the untreated, GP, GPHCF, and GPHCFD hydrogel treatment groups were subjected to Western blotting. **(I)** A heatmap was used to statistically assess the levels of CCR2, p-IRE1, and XBP1 protein expression according to Western blotting data. *n* = 5 per team. *P < 0.05, *NS*: non-significant. The data is displayed as mean ± SD.

The GO enrichment analysis bubble chart shows that genes significantly downregulated in local NK cells treated with the GPHCFD hydrogel in diabetic wound tissue are enriched in the following functional pathways: negative regulation of natural killer cell differentiation, ERS-related functions (PERK complex, response to unfolded proteins, and endoplasmic reticulum membrane) (Fig. 6A). Volcano plot analysis revealed that the expression of NK cell CCR2, ERS-related genes XBP1, ATF6B, IRE1, PERK, ATF6, ATF4, and inflammatory factors TNF-α and IFN-γ was significantly downregulated (Fig. 6B). To further validate the GPHCFD hydrogel’s impact on NK cells in diabetic wounds, we used flow cytometry to detect NK cells in diabetic wounds on day 3 in the untreated, GP, GPHCF, and GPHCFD groups—specifically CD45^+^CD3^−^NK1.1^+^ cells. The GPHCFD group had the lowest percentage of CD3^−^NK1.1^+^cells among the CD45 + cells when compared to the other three groups. This suggests that while the NK cells in the other three groups displayed significant abnormal proliferation, the GPHCFD group showed only mild abnormal NK cell proliferation (Fig. 6C, E). The proportion of CD11b^+^CD27^+^ NK cells among CD3^−^NK1.1^+^cells in diabetic wound sites increased most significantly among the four groups in the GPHCFD group (Fig. 6C, F), but the percentage of CD11b^+^CD27^−^NK cells among CD3^−^NK1.1^+^ cells did not significantly alter (Fig. 6C, G). The GPHCFD hydrogel treatment significantly enhanced NK cell maturation toward the CD11b^+^CD27^+^ NK cells. The NK cells from diabetic wound sites in the untreated, GP, GPHCF, and GPHCFD groups were then subjected to Western blotting analysis. The expression levels of CCR2 and ERS-related proteins p-IRE1 and XBP1 were lowest in the wound-site NK cells of the GPHCFD hydrogel treatment group (Fig. 6H, I, S8). These results demonstrated that the GPHCFD hydrogel might inhibit NK cell CCR2 expression, reduce excessive ERS levels, suppress abnormal NK cell proliferation and inflammatory factor secretion, and promote NK cell maturation. Mature NK cells can regulate wound inflammation homeostasis, increase re-epithelialization speed, and improve collagen deposition [38].

### The GPHCFD hydrogel promoted M1–M2 macrophage polarization by inhibiting ERS in macrophages

**Fig. 7:**
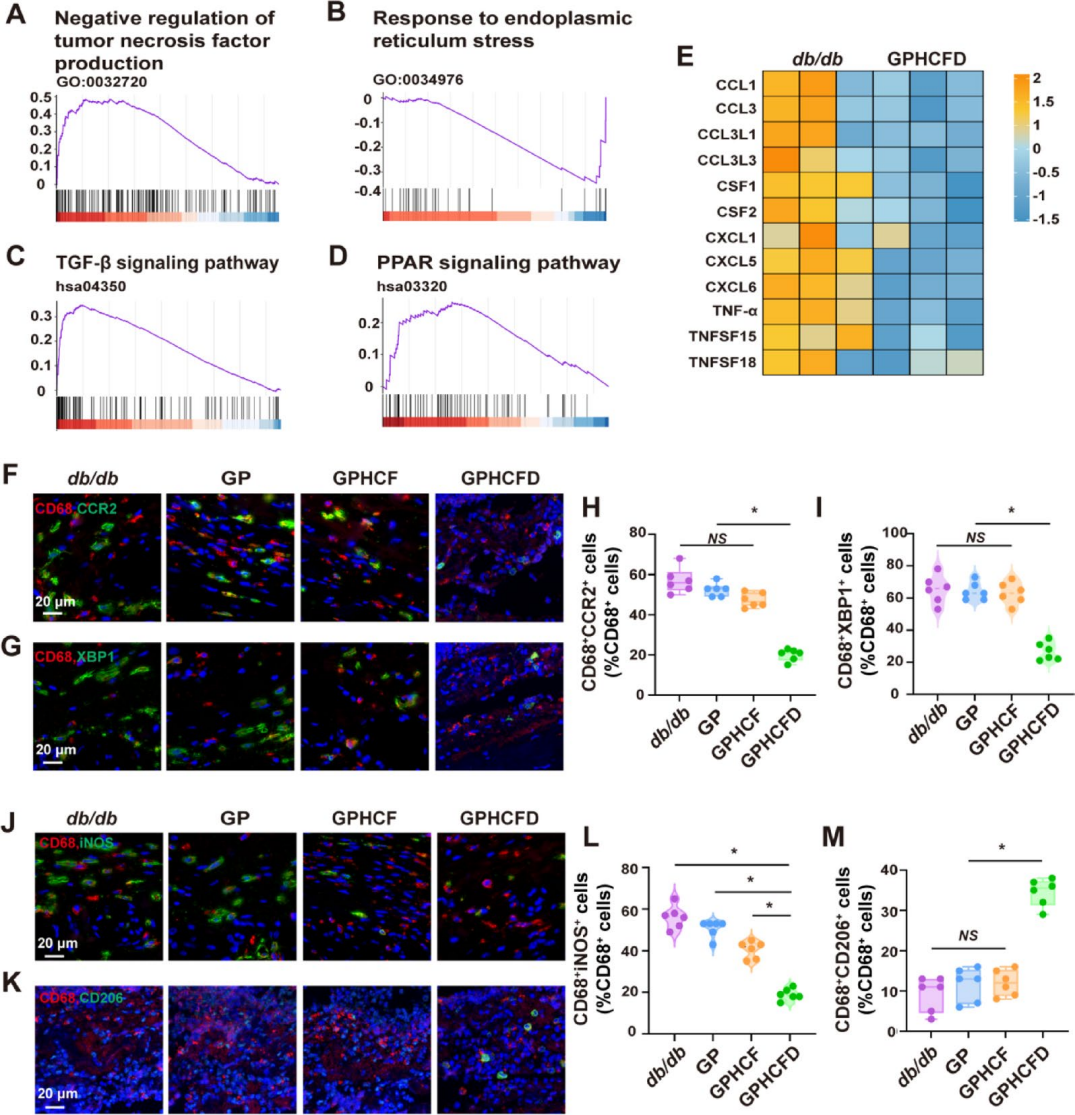
The GPHCFD hydrogel promotes macrophage polarization toward the M2 type by inhibiting ERS in macrophages. **(A)** GSEA plot analysis of gene enrichment related to the negative regulation of tumor necrosis factor production function in local macrophages in diabetic wound tissue in the GPHCFD group. **(B)** The TGF-β signaling pathway in macrophages in the GPHCFD group was analyzed using a GSEA plot. **(C)** GSEA plot analysis of gene enrichment related to the response to ERS function in macrophages in the GPHCFD group. *n* = 3 per group. **(D)** GSEA plot analysis of gene enrichment related to the KEGG PPAR signaling pathway in macrophages in the GPHCFD group. *n* = 3 per group. **(E)** Heatmap analysis of inflammatory factor expression in local macrophages from the untreated diabetic wound group and the GPHCFD hydrogel-treated group. *n* = 3 per group. **(F)** Immunofluorescence detection of CD68^+^CCR2^+^ cells in diabetic wounds in the untreated, GP, GPHCF, and GPHCFD hydrogel-treated groups. Scale bar, 20 μm. **(G)** Identification of CD68^+^XBP1^+^ cells in the diabetic wounds in the four groups by immunofluorescence. 20 μm Scale bar. **(H)** The percentage of CD68^+^CCR2^+^ cells among CD68^+^ cells is statistically analyzed. *n* = 6 each group. *P < 0.05, *NS*: non-significant. **(I)** Statistical analysis of the proportion of CD68^+^XBP1^+^ cells among CD68^+^ cells. *n* = 6 in each group. **(J)** Immunofluorescence detection of CD68^+^ iNOS^+^ cells in the diabetic wounds in the four groups. Scale bar, 20 μm. **(K)** Immunofluorescence detection of CD68^+^CD206^+^ cells in the diabetic wounds in the four groups. 20 μm Scale bar. **(L)** The percentage of CD68^+^iNOS^+^ cells among CD68^+^ cells is statistically analyzed. **(M)** Statistical analysis of the proportion of CD68^+^CD206^+^ cells among CD68^+^ cells. *n* = 6 per team. *P < 0.05, *NS*: non-significant. The data is displayed as mean ± SD.

To further explore the effects of the GPHCFD hydrogel on local macrophages in diabetic wounds, we analyzed macrophage data from the untreated and GPHCFD groups using single-cell sequencing. GSEA analysis showed that in the diabetic wound tissue of the GPHCFD group, the negatively regulated genes related to tumor necrosis factor production (inhibiting macrophage polarization to M1) were significantly upregulated in local macrophages (Fig. 7A). The response-related genes of ERS were significantly downregulated (Fig. 7B), while the KEGG pathway that promotes macrophage polarization towards the M2 type, namely the TGF-β signaling pathway-related genes, was significantly upregulated (Fig. 7C). The KEGG PPAR signal pathway-related genes that interact with ERS and macrophage polarization to M2 type were significantly up-regulated, further indicating that the GPHCFD hydrogel can promote macrophage polarization to M2 type by regulating ERS (Fig. 7D). The heatmap analysis revealed that compared with those in untreated diabetic wound tissue, the levels of inflammatory factors CCL1, CCL3, CCL3L1, CCL3L3, CSF1, CSF2, CXCL1, CXCL15, CXCL6, TNF-α, TNFSF15, and TNFSF18 were significantly decreased in macrophages in the GPHCFD group (Fig. 7E). CD68 is a characteristic marker of macrophages; thus, we used immunofluorescence to assess the level of CCR2, the ERS biomarker XBP1, the M1 signature iNOS, and the M2 biomarker CD206 in CD68^+^ cells in diabetic wound tissues from the untreated, GP, GPHCF, and GPHCFD groups. The proportions of CD68^+^CCR2^+^ and CD68^+^XBP1^+^ cells were significantly reduced (Fig. 7F-I), indicating that the GPHCFD hydrogel can inhibit macrophage CCR2 expression and reduce macrophage ERS. The proportion of CD68^+^iNOS^+^ cells significantly decreased, while that of CD68^+^CD206^+^ cells significantly increased, indicating that the GPHCFD hydrogel promotes the M1–M2 conversion of diabetic wound macrophages (Fig. 7J-M). The above results demonstrated that the GPHCFD hydrogel might inhibit the expression of CCR2 in local macrophages at diabetic wound sites, reduce excessive ERS in macrophages, and promote macrophage M1–M2 conversion.

## Discussion

Here, we designed an intelligent nanocomposite hydrogel system, GPHCFD, to autonomously sense ERS overload states, release targeted therapeutic plasmids on demand, and break the MCP-1-driven inflammation and dysfunction cycle through gene silencing. We induced excessive ERS in fibroblasts in vitro using Tg. Fibroblasts were treated with either MCP-1-shRNA plasmid or scr-shRNA plasmid (scrambled short hairpin RNA plasmid) in combination with GPHCF hydrogel. Compared to the GPHCF-scr-shRNA hydrogel group, the PBS group, and the untreated Tg-induced fibroblast group, MCP-1 gene expression was effectively knocked down in the GPHCF-MCP-1-shRNA (GPHCFD) hydrogel group (Fig. S9A). No significant differences in expression of the non-target genes β-actin and GAPDH were observed between the GPHCF-MCP-1-shRNA hydrogel group and the GPHCF-scr-shRNA hydrogel group, indicating that MCP-1 knockdown induced by MCP-1-shRNA in the GPHCF-MCP-1-shRNA hydrogel did not produce off-target effects (Fig. S9B, C). We further observed that the relative proliferation rates of fibroblasts treated with extracts from GP, GPHCF, GPHCF-MCP-1-shRNA, and GPHCF-scr-shRNA hydrogels, as well as MCP-1-shRNA plasmid solution, exceeded 80% at both 24 and 48 h, surpassing the ISO 10993-5 standard cytotoxicity threshold (70%). demonstrating the non-cytotoxicity of GPHCF-MCP-1-shRNA (Fig. S10). These results demonstrated that MCP-1-shRNA in GPHCFD hydrogels exhibited no off-target effects and that GPHCFD hydrogels are non-cytotoxic, both of which were beneficial for wound healing.

Additionally, FAP1’s net positive charge slightly enhances the overall positive charge of His–CTs; its arginine residues provide a locally high positive charge density, enhancing DNA phosphate backbone binding [39, 40]. Furthermore, its hydrophobic residues promote DNA condensation through hydrophobic interactions, forming more compact nanospheres [41]. These findings suggested that FAP1 might contribute to the formation of nanoparticle complexes between plasmids and HF.

Based on rheological characterization results, GPHCFD hydrogel exhibits significant shear thinning behavior (η maintained at 7–20 mPa·s), conferring excellent injectability and enabling minimally invasive, precise delivery via ultra-fine needles, thereby reducing tissue damage around diabetic wounds [42]. The rapid and controllable curing properties (50–80 s) and stable elastic network of the GPHCFD hydrogel (G’ > G’’) form a mechanical barrier at the wound site, effectively blocking pathogens and locking in bioactive factors, while providing a temporary scaffold for cell migration [43]. The storage modulus of the GPHCFD hydrogel (G’ < 1000 Pa) is close to the mechanical properties of soft tissues [44], which is beneficial for wound healing. The dynamic borate ester bond-mediated self-healing capability ensures that the hydrogel maintains structural integrity and nanoparticle sustained-release functionality in active site wounds, addressing the clinical challenge of traditional scaffolds prone to displacement and rupture [45]. Therefore, the GPHCFD hydrogels exhibited superior rheological properties, rendering them clinically viable for diabetic wound management.

By reducing MCP-1 expression levels, the hydrogel decreased CCR2 expression in macrophages and NK cells and alleviated excessive ERS, thereby indirectly improving their functional capacity. The pro-inflammatory M1-type of macrophages, which strongly expressed TNF-α, IL-1β, and iNOS, are transformed into the reparative M2-type, which strongly expressed Arg1 and CD206, by the GPHCFD hydrogel. Notably, in the GPHCFD hydrogel-treated co-culture system of fibroblasts and macrophages, exogenous supplementation with recombinant MCP-1 protein led to elevated levels of ERS in macrophages, shifting their polarization toward the pro-inflammatory M1 phenotype characterized by TNF-α expression (Fig. S11A-C). In stark contrast, when a CCR2-specific antagonist was added to the hydrogel-treated fibroblast-macrophage co-culture system, macrophage ERS levels significantly decreased, and they tended to express the anti-inflammatory M2 phenotype characterized by Arg1 expression (Fig. S11A-C). These results indicated that the GPHCFD hydrogels might exert their effects on macrophages via the MCP-1/CCR2 pathway. This hydrogel inhibited abnormal proliferation and CCR2 expression in CD45^+^CD3^−^NK1.1^+^ natural killer cells, resolved maturation defects in CD11b^−^CD45^+^CD3^−^NK1.1^+^ natural killer cells, and suppressed secretion of inflammatory molecules such as IFN-γ and TNF-α induced by excessive ERS. Improving the aforementioned functions of NK cells promoted wound re-epithelialization and inhibited excessive inflammation [38]. These results showed that the GPHCFD hydrogel overcame the drawbacks of current approaches, which usually targeted a single cell type, by concurrently addressing ERS in fibroblasts, macrophages, and NK cells. This approach achieved multicell synergistic regulation, leading to systematic wound-healing microenvironment repair.

This study developed the GPHCFD nanocomposite hydrogels that synergistically promoted diabetic wound healing through targeted MCP-1 gene editing and immune reprogramming, yet limitations remain. Verification of the off-target effects of the GPHCFD hydrogels remains insufficient. Future work could involve constructing an shRNA-resistant MCP-1 overexpression plasmid and combining it with GPHCF to prepare a hydrogel for application to fibroblasts. This would allow observation of changes in ERS and proliferation/migration functions within fibroblasts, thereby strengthening validation of the off-target effects of the GPHCFD hydrogels. Furthermore, existing NK cell lines (including NK-92, NK101, and NK3.3) and primary NK cells isolated from mouse spleen tissue exhibit different phenotypes and differentiation patterns compared to NK cells at diabetic wound sites. This made it challenging to simulate the fibroblast-NK cell interaction model in vitro. Future studies should strengthen validation by supplementing MCP-1 protein and CCR2 inhibitors in vivo to enhance the functional effects of the GPHCFD nanocomposite hydrogels on improving NK cell function through MCP-1/CCR2 inhibition. This aims to strengthen the validation of how the GPHCFD nanocomposite hydrogels effectively enhance NK cell function by suppressing the MCP-1/CCR2 pathway. Furthermore, experiments revealed that while the GPHCFD hydrogels enhance the function of fibroblasts, macrophages, and NK cells in diabetic wounds, GPHCF also affects these three cell types. This may relate to the local wound effects of FAP1, CTs, and Gelma, warranting further validation in future studies. Furthermore, the following aspects warrant future exploration: Response release efficiency may be disrupted by complex wound microenvironments; the long-term safety and cell specificity of the immune regulation mechanism require further validation; clinical translation faces challenges in delivery systems and scaling.

## Conclusion

In summary, we have successfully created a hydrogel system for the intelligent nanocomposite GPHCFD that responds to ERS in this study. The GPHCFD hydrogel can precisely sense and respond to excessive ERS in the microenvironment of diabetic wounds, enabling the triggered release of plasmids. Through the FAP1 peptide, the nanocarrier actively targets key effector cells in the wound—fibroblasts—delivering the pGPU6/GFP/Neo MCP-1–shRNA plasmid to efficiently and stably knock out the MCP-1 gene, effectively inhibiting excessive ERS in fibroblasts and significantly restoring their proliferation, migration, and collagen secretion capabilities. These effects achieve fibroblast-specific functional reprogramming, fundamentally improving the foundation for tissue regeneration. Due to immune microenvironment co-reprogramming, MCP-1-knockout fibroblasts significantly reduce abnormal macrophage and NK cell CCR2 receptor activation, lowering the ERS levels of these immune cells and synergistically improving the abnormal proliferation, maturation defects, and excessive secretion of inflammatory factors in NK cells. Furthermore, the hydrogel enhances the ability of macrophages to polarize toward the pro-repair M2 type, creating an immune microenvironment conducive to healing. This study innovatively integrates three functional modules, namely, ERS-responsive drug delivery, fibroblast-specific gene editing, and systemic immune microenvironment regulation, into a single hydrogel platform, addressing the limitations of existing diabetic wound dressings with single functionalities. This new tissue regeneration–immune regulation synergistic paradigm significantly improves the efficiency and quality of diabetic wound healing and provides important design concepts and theoretical and practical foundations for developing next-generation multifunctional smart wound dressings.

## Materials and methods

### Preparation of HCFD nanoparticles

MCP-1-targeted shRNAs were obtained from GenePharm Technology (Shanghai, China). Stable *Escherichia coli* culturing was performed using the pGPU6/GFP/Neo MCP-1 plasmid (transformed with DH5α-shRNA), which encodes shRNA and targets MCP-1 mRNA. For activation, 10-(N-Morpholino)ethanesulfonic acid) buffer (MES) 20 mM (pH 6.0) was used to dissolve His. NHS (N-hydroxy succinimide) and 1-ethyl-3-(3-dimethylaminopropyl)carbodiimide (EDC) were added at a molar proportion of 2:1:1 for EDC: NHS: His. To create the active ester intermediate, the mixture was agitated for half an hour. At a molar ratio of 1:5, CTs (1% w/v, dissolved in 0.1 M acetic acid buffer, pH 5.0) were added to the activated solution. After bringing the pH down to 6.0, the combination was allowed to react for a full day. The reaction mixture was dialyzed to remove unreacted histidine and then freeze-dried to obtain the His–CTs complex. PBS with a pH of 7.0 was used to dissolve the His–CTs combination. Sulfosuccinimidyl -(N-maleimidomethyl)cyclohexane-1-carboxylate, a maleimide-containing bifunctional crosslinker, was added in excess at a molar ratio of 15:1 Sulfo–SMCC: His–CTs. The mixture was then reacted for 2 h and dialyzed to remove unreacted reagent, yielding maleimide-activated HC. The maleimide-activated His–CTs was subsequently mixed with FAP1 peptide at a molar ratio of FAP1:maleimide group of 1.2:1 and reacted for 4 h. To remove any remaining maleimide groups, cysteine was added at a 10% (in relation to FAP1) molar ratio. The mixture was then allowed to react for ten minutes at room temperature. To obtain HCF, the product was freeze-dried. 0.8 mg/mL of pDNA plasmid was combined with 1 mg/mL of HCF solution. HCF: pDNA complexes were prepared at a 10:1 N/P ratio to obtain HCFD nanoparticles.

The morphologies of CTS, CTS-pDNA, HC-pDNA, and HCFD complexes were tested using DLS, SEM, and TEM. Using the Zeta sizer Nano ZS, the complexes’ zeta potentials were instantly determined.

### GPHCFD hydrogel Preparation

First, MES buffer (pH 5.5) was used to dissolve PBA. EDC and NHS were added to the mixture to activate the carboxyl groups, and it was stirred for 30 min at room temperature. A GelMA solution made with PBS (pH 7.4) was mixed with the activated solution. The reaction was executed for 2 h at room temperature, then dialysis to get rid of the unreacted PBA. The product was then freeze-dried to obtain the GP complex. A mixture containing 10% (w/v) GelMA dissolved in PBS, 5% (w/v) PEG 20 kDa as a pore-forming agent, and 1 mM 4-carboxyphenylboronic acid was pregrafted onto the amino groups of GelMA, along with 0.5% (w/v) photoinitiator LAP. In order to create a primary gel network with PEG intercalated, the samples were first properly mixed and dissolved before being subjected to UV radiation (365 nm, 5 mW/cm^2^) for 60 s. The gel was subsequently immersed in PBS and shaken for 48 h to completely dissolve and release the PEG, forming large pores. HCFD nanoparticles were placed in pH 9.0 borate buffer to promote borate ester bond formation. A solution containing 1 mg/mL of PBA-GelMA hydrogel with macroporous pores was immersed in the complex solution. The reaction was performed for 2 h. The mixture was then transferred to PBS (pH 7.4), and 10 mM His was added for stabilization.

CTs was labeled with FITC–NHS at a 1:50 ratio in the dark for 4 h prior to nanoparticle synthesis and purified by dialysis. The GPHCFD hydrogels were further prepared, and HCFD nanoparticles in the hydrogels were detected using fluorescence confocal microscopy. The morphology of the prepared liquid gel, GP, GPHCF, and GPHCFD hydrogels was examined by AFM, and the distribution of nanoparticle diameters was determined.

### Cell culture

Qingqi (Shanghai) Biotechnology Development Co., Ltd. supplied the human skin fibroblasts, which were then cultivated in high glucose DMEM medium (Dulbecco’s modified Eagle’s medium; Gibco) enhanced with 10% FBS (extracellular vesicle-free fetal bovine serum; Gibco) and 1% Penicillin-streptomycin (Gibco). Sunncell Biotechnology Co., Ltd. was the supplier of the THP-1 human monocytic cell line. Under the same incubation conditions, these cells were kept in Roswell Park Memorial Institute (RPMI) 1640 media (Gibco) supplemented with 10% FBS and 1% penicillin-streptomycin. In accordance with the manufacturer’s instructions, ethidium homodimer-1 was used to induce red fluorescence from dead fibroblasts, and Calcein AM was used to stain live fibroblasts. Fibroblasts were cocultured with the GPHCFD hydrogel after the samples were incubated for 20 min. Subsequently, 2D and 3D images were captured using fluorescence confocal imaging.

### Fibroblast proliferation assay

After being seeded, fibroblasts (2 × 10^4^) were exposed with 100 nM Tg for six hours. After that, the cells were separated into five groups, cocultured for one, three, five, and seven days using the Gel, GP, GPHC, GPHCF, and GPHCFD hydrogels, and dyed with CFDA–SE (ThermoFisher Scientific) in accordance with the guidelines provided by the manufacturer. The five hydrogels were used to assess fibroblast proliferation using the CCK-8 assay. The supernatant was disposed of following 1, 3, 5, or 7 days, and each well received 100 µL of CCK-8 solution. After that, the cells were cultured for four more hours. The OD value for each well was measured at 450 nm using the ELISA reader (Infinite M200).

### Cell migration assay

A wound-healing assay was used to quantify cell migration in a six-well Transwell plate (Corning Incorporated). Fibroblasts were serum-starved for the whole night following a 24-hour cell attachment period. Fibroblasts were treated with 100 nM Tg for six hours in order to mimic ERS, and they were then cultivated till > 90% attached to the cells. Following a PBS wash that left a 0.5 cm gap, the cells were separated into four groups, three of which were cocultured with the hydrogels for control, GP, GPHCF, and GPHCFD. Images of wound closure were taken at 12, 24, and 36 h. To determine the percentage of in vitro wound closure, the area of the wound was measured by ImageJ software.

### Construction and culture of ERS reporter cell lines

The fibroblasts were acquired from Qingqi (Shanghai) Biotechnology Development Co., Ltd. and cultivated in high-glucose DMEM medium and 10% extracellular vesicle-free FBS was added. Sunncell Biotechnology Co., Ltd. was the supplier of the THP-1 human monocytic cell line. Three tandem ERSE cis-response elements (CTCGAGACAGGTGCTGACGTGGCGATTC) were inserted into the pmCherry-1 vector (Clontech) via standard PCR cloning, replacing the native promoter to drive mCherry expression. The reporter gene vector was transfected into THP-1 cells and fibroblasts using Lipofectamine 2000 (Thermo Fisher Scientific), in accordance with the manufacturer’s instructions. The selecting agent blasticidin (Invivogen) produced cells that consistently expressed the reporter gene.

Two cell types were cocultured in a Transwell coculture dish at a 1:1 ratio. The lower layer contained fibroblasts. The upper layer contained THP-1 cells induced by 20 ng/mL PMA for 48 h to obtain M0 macrophages, which were then cultured in serum-free medium (Cyagen Biosciences, China). To cause increased ERS, cells were exposed to 100 nM Tg for six hours. The following groups were created from the cells: the PBS group without Tg induction, the Tg-induced fibroblast and macrophage group, the Tg-induced macrophage group, the GP hydrogel-treated Tg-induced fibroblast and macrophage group, the GPHCF hydrogel-treated Tg-induced fibroblast and macrophage group, and the GPHCFD hydrogel-treated Tg-induced fibroblast and macrophage group. The aforementioned hydrogels were placed in the lower layer of the coculture dish. The samples were then prepared for subsequent flow cytometry and Western blotting analyses.

### Flow cytometry

Fluorescently labeled antibodies, such as, FITC anti-mouse CD11b (Abcam, 1:300), PE anti-mouse mCherry (Abcam, 1:300), APC-Cy7 anti-mouse CD45 antibody (Abcam, 1:200), and Pacific blue anti-mouse CD3 antibody, were added to the cells after they had been cleaned. As directed by the manufacturer (BD Biosciences), apply the following antibodies for protein molecular staining: PE-Cy7 anti-mouse NK1.1 antibody (Abcam, 1:200), PE anti-mouse CD11b antibody (Abcam, 1:200), and AmCyan anti-mouse CD27 antibody (Abcam, 1:200). After being cleaned and resuspended in PBS, a BD Biosciences BD FACS flow cytometer was used to analyze the cells. The data was analyzed using FlowJo software (version 7.6.1) from TreeStar Co., Ltd.

### Animals

Chongqing Ensvell Laboratory Animal Sales Co., Ltd. in China provided the male *db/db* mice.A biopsy punch (diameter 1.0 cm) was used to remove the epidermis and dermis from the dorsal surface. Four groups of mice were given different treatments for their dorsal wounds: untreated, the GP hydrogel treatment, the GPHCF hydrogel treatment, and the GPHCFD hydrogel treatment. Using photos taken on day 0, 5, 10, and 15, wound closure was evaluated and examined using ImageJ software.

### Histological analysis and Immunofluorescence assay

On days eight and fifteen, the mice were put to death. 10% formalin was used to fix the wound tissue, and paraffin was regularly used to embed it. Masson’s trichrome and hematoxylin and eosin (H&E) were utilized for staining diabetic wound sections. ServiceBio scanned the images. Collagen and dermis thickness were measured by ImageJ v1.8.0.

Wound slices were dewaxed and washed with PBS in preparation for immunofluorescence staining. To recover the antigen, tissue slices were treated with sodium citrate buffer for ten minutes at 95°C. This was followed by 10 min of penetration with 0.1% Triton X-100. Following blocking, anti-mouse CHOP (Abcam, 1:300), anti-DCN (Abcam, 1:100), anti-mouse CD68 (Abcam, 1:100), anti-mouse CCR2 (Abcam, 1:100), anti-mouse CD206 (Abcam, 1:100), or anti-mouse iNOS (Abcam, 1:100) antibodies were added to tissue sections incubated at 4°C for 8 h using Tween 20 containing 2% BSA. After adding secondary goat anti-mouse (Abcam, 1:200) and rabbit anti-mouse (Abcam, 1:400) antibodies, the samples were incubated for two hours at room temperature. A 20× magnified confocal fluorescence microscope (Leica SPII) was used to capture fluorescent pictures.

### Single-cell sequencing

To explore ERS transmission between cells in wound tissue, wound tissues were preserved in a sterile tissue preservation solution immediately after sampling during surgery. The samples were then sent to Hangzhou Lianchuan Biotechnology Co., Ltd. for single-cell isolation and single-cell sequencing within 48 h post-sampling. The same company performed the data analyses.

### Flow cytometry sorting

Wound tissue was taken from *db/db* mice on day 3 after injury and washed with PBS containing two antibodies. Observing the guidelines provided by the manufacturer for the Epidermis Dissociation Kit (Miltenyi Biotec), the tissue was sliced into tiny pieces and the dissociation solution was made. The wound tissue samples from *db/db* mice were then digested and separated. NK cells were isolated in accordance with the guidelines supplied by Miltenyi Biotec, the manufacturer of the NK Cell Isolation Kit.

### Western blotting

SDS-PAGE was used to extract and separate the proteins from cell lysates on a 10–12% polyacrylamide gel. The proteins were moved from the polyacrylamide gel to a PVDF membrane following electrophoresis. After that, primary antibodies, including anti-CHOP, were incubated on the membrane. The antibodies are as follows: anti-MCP-1 (Abcam, ab216484; 1:2000), anti-α-tubulin (Abcam, ab6160; 1:2000), anti-CCR2 (Abcam, ab216839; 1:2000), anti-phosphorylated IRE1α (Abcam, ab48187; 1:1000), and anti-XBP1 (Abcam, ab229912; 1:2000).

### Quantitative real-time PCR

The cells were treated with Invitrogen’s TRIzol Reagent. A QuantiTect Reverse Transcription Kit (Qiagen) was used to extract total RNA and reverse-transcribe it into cDNA in accordance with the manufacturer’s instructions.The PCR was performed using the SYBR Green qPCR Master Mix (MedChem). The threshold cycle (CT) values of the housekeeping gene and target genes were utilized to calculate the gene expression levels in each sample using the 2 ^− △△^ CT approach. The primers for the target genes (Table S1) and the housekeeping gene (GAPDH) are in Additional File 1.

### Statistical analyses

The statistical analyses were conducted using SPSS (version 29.0; IBM SPSS, Armonk, NY) and GraphPad Prism (version 10.3.0; GraphPad Software). While a paired t test was used to evaluate differences between paired groups, the Mann-Whitney U test was utilized to examine clear differences between two unpaired groups.When comparing three or more groups, the Bonferroni post hoc test was employed together with one-, two-, or repeated-measures analysis of variance (ANOVA).

## Supplementary Information

Supplementary Material 1

## Data Availability

All of the information is contained in the major text and the supplemental materials.
