# Supplementary material for: An endoplasmic reticulum stress-responsive nanocomposite hydrogel for diabetic wound healing through a fibroblast-immune cell dual regulation hub
Source: J Nanobiotechnology. 2025 Oct 24;23:689. doi: 10.1186/s12951-025-03732-0 (PMC12551225; doi:10.1186/s12951-025-03732-0)
Supplement: Supplementary file 1 — Supplementary Material 1 [file 12951_2025_3732_MOESM1_ESM.docx]

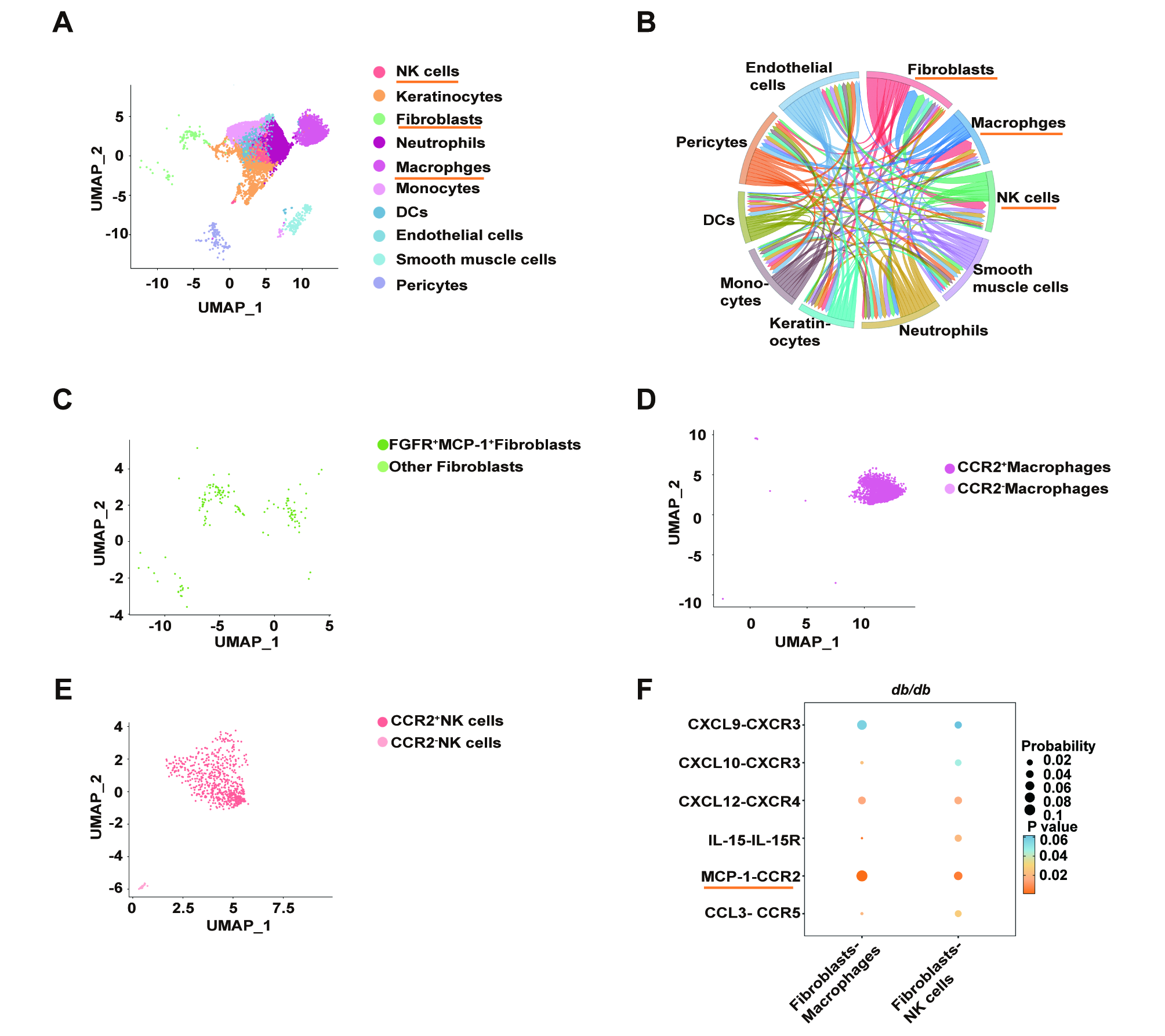


Fig. S1. Single-cell sequencing analysis of diabetic wound tissue

**A.** UMAP plot analysis of 10 cell clusters in the diabetic wound group treated with hydrogel. **B.** String plot analysis revealed significant interactions between fibroblasts, NK cells, and macrophages in diabetic wound tissue. **C.** UMAP plot analysis showed that fibroblasts in diabetic wound tissue exhibited high expression of EGFR and MCP-1. **D.** UMAP plot analysis revealed that macrophages in diabetic wound tissue locally overexpress CCR2. **E.** UMAP plot analysis revealed that NK cells in diabetic wound tissue locally overexpress CCR2. **F.** Molecular interaction bubble plot analysis revealed that among the interactions between fibroblasts, macrophages, and NK cells in diabetic wound tissue, the MCP-1-CCR2 interaction was the most significant. n=3 per group.


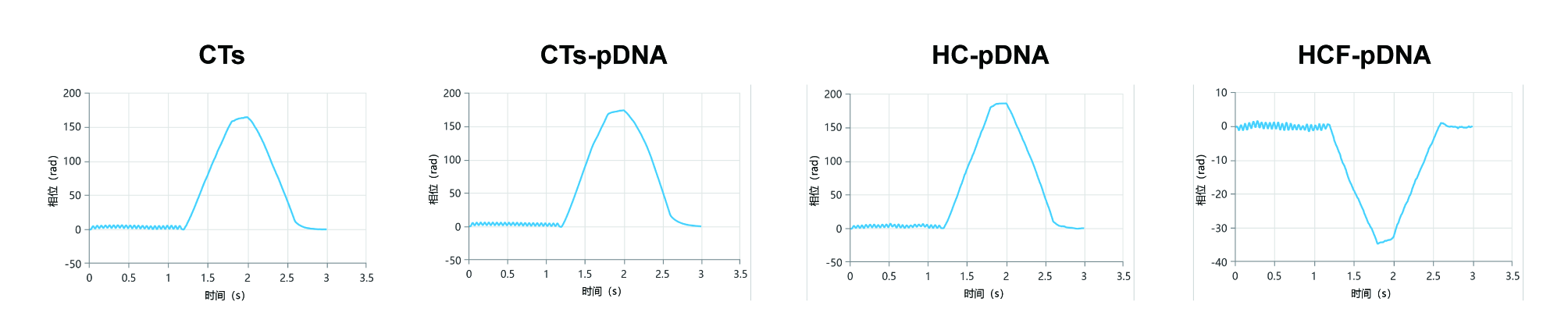


Fig. S2. Phase diagram analysis of HCFD nanoparticle synthesis

The phase diagram shows that, compared with CTs and HC, HCF is most likely to form nanoparticles with plasmids.


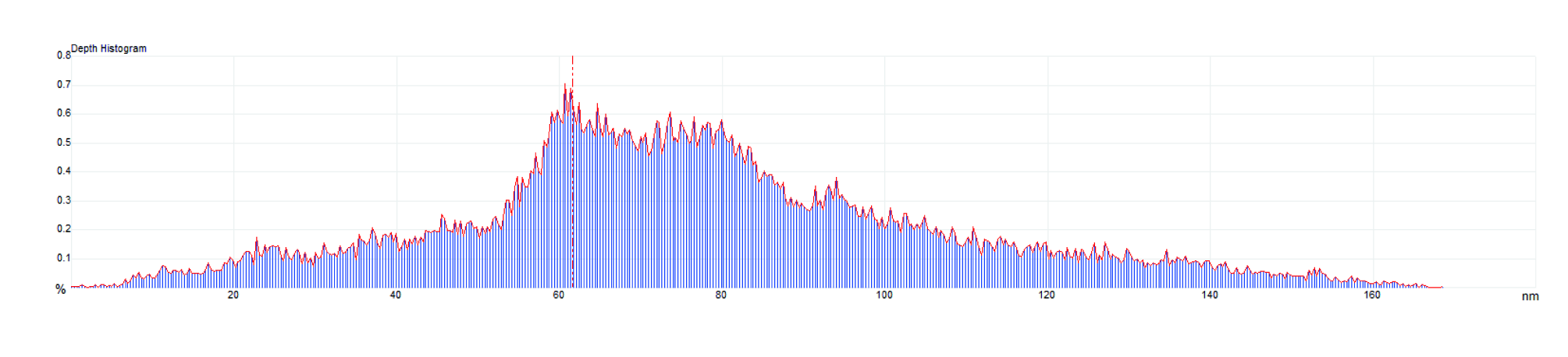


Fig. S3. AFM analysis of the particle size of HCFD nanoparticles in liquid hydrogels

Particle size analysis shows that the particle size of HCFD nanoparticles in liquid hydrogels is mainly between 50 and 90 nm.


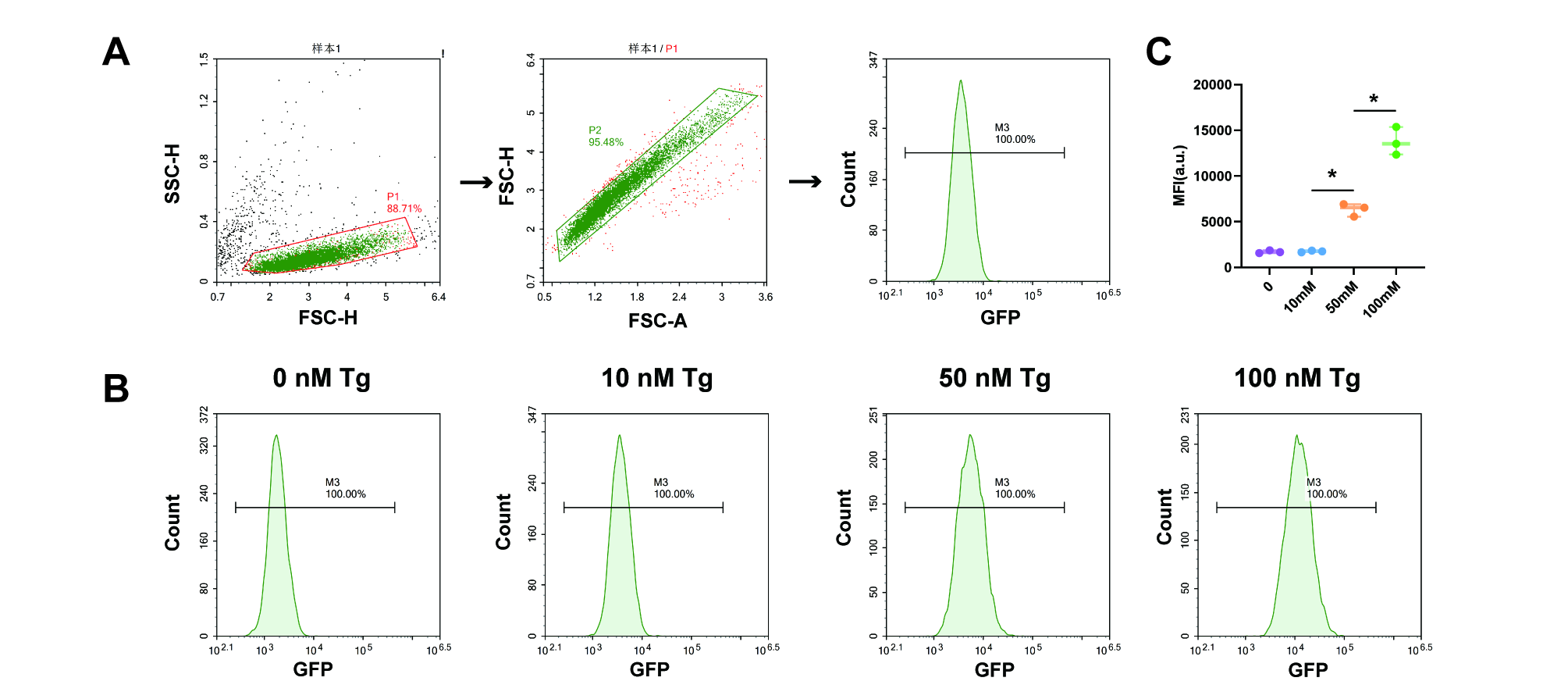


Fig. S4. Flow cytometry detection of fibroblast plasmid transfection levels

1. **B.** Induce the ERS in fibroblasts using Tg concentrations of 0 mM, 10 mM, 50 mM, and 100 mM.

Using flow cytometry, assess the fluorescence intensity of cells transfected with a GFP-labeled

plasmid. **C.** Statistical analysis indicates that as Tg concentrations (ERS level) increases in fibroblasts, plasmid transfection efficiency is enhanced. n=3 per team. **P*<0.05. The data is displayed as mean±SD.


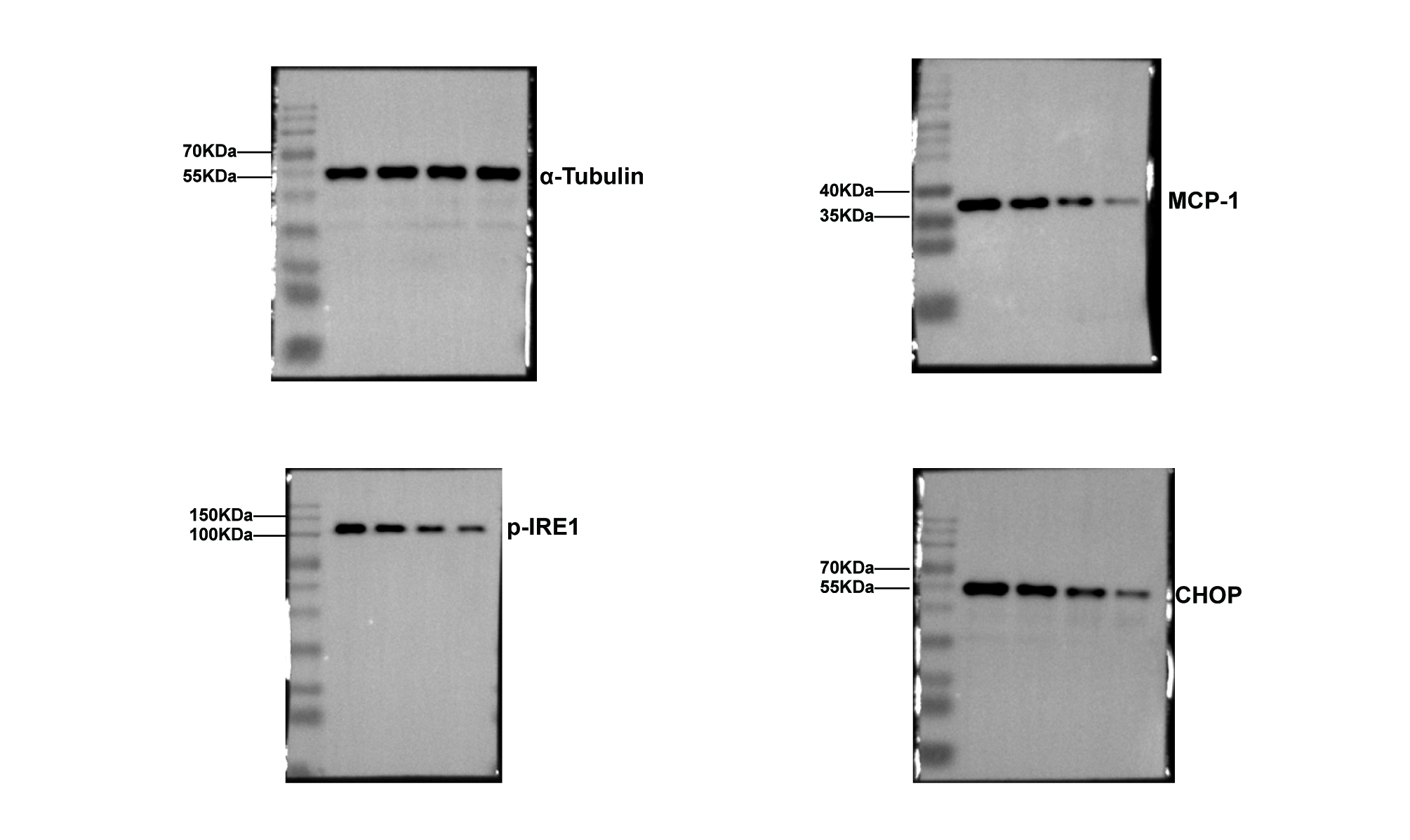


Fig. S5. Western blot full-membrane analysis of MCP-1, p-IRE1, and CHOP in fibroblasts in the four groups: non-hydrogel group, GP, GPHCF, and GPHCFD hydrogel groups.


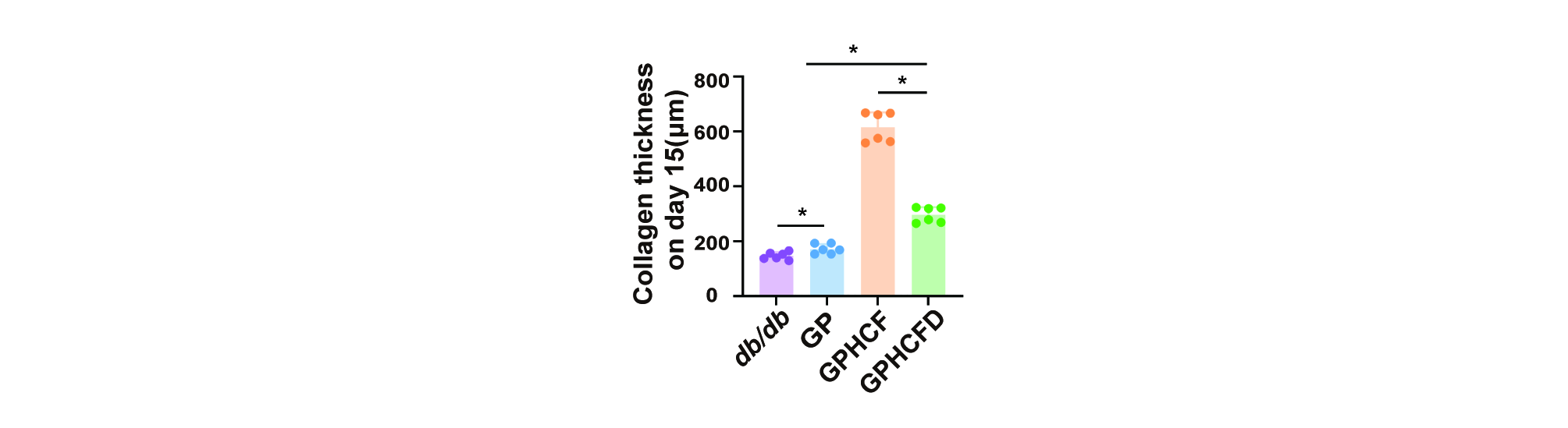


Fig. S6. Statistical analysis of collagen thickness in diabetic wounds treated with the non-hydrogel group, GP, GPHCF, and GPHCFD hydrogel groups on day 15. n=6 per team. **P*<0.05. The data is displayed as mean±SD.


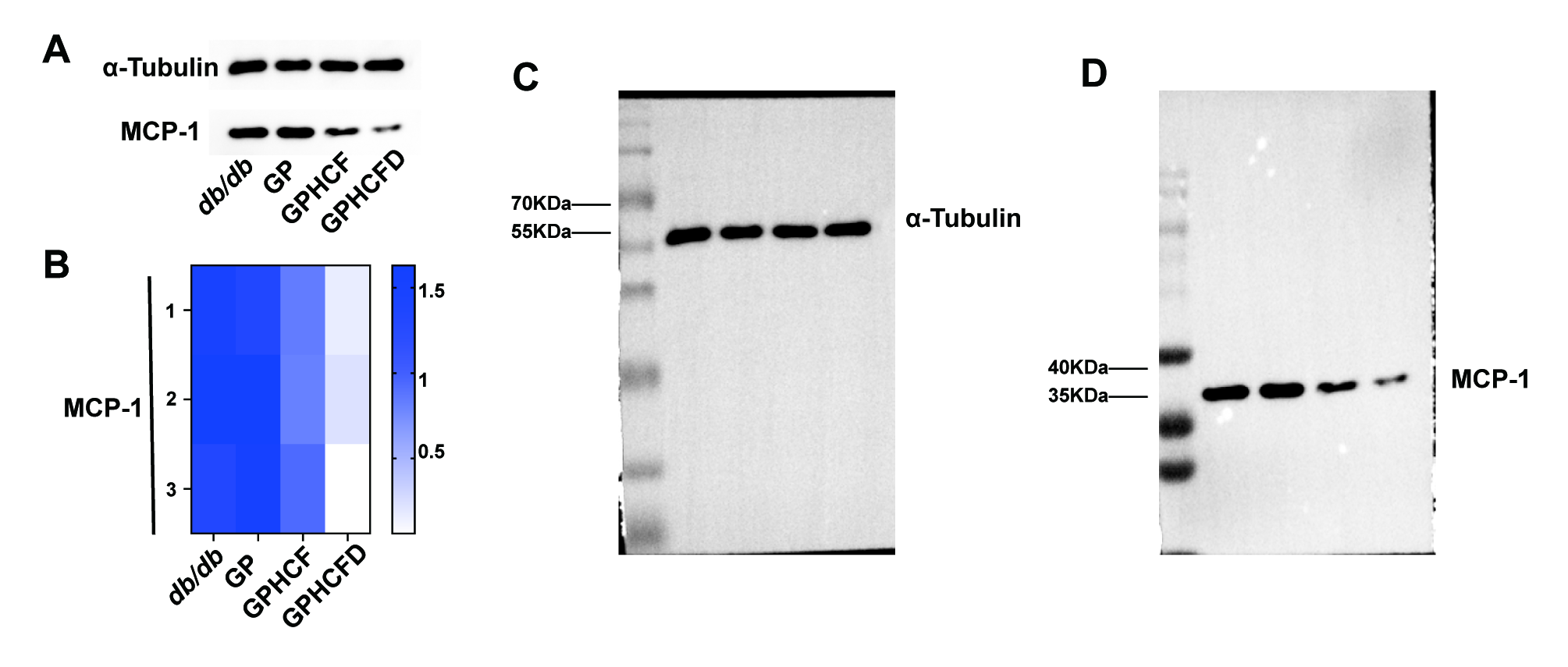


Fig. S7. Western blot analysis of local MCP-1 expression in diabetic wound tissue treated with GPHCFD hydrogel.

1. Western blot analysis was performed to detect the expression levels of MCP-1 in wound tissue from the non-hydrogel group and the GP, GPHCF, and GPHCFD hydrogel groups. **B.** Heatmap statistics of MCP-1 expression levels in four groups of wound tissue sections. **C.** Western blot full-membrane analysis of α-Tubulin. D. Western blot full-membrane analysis of MCP-1.


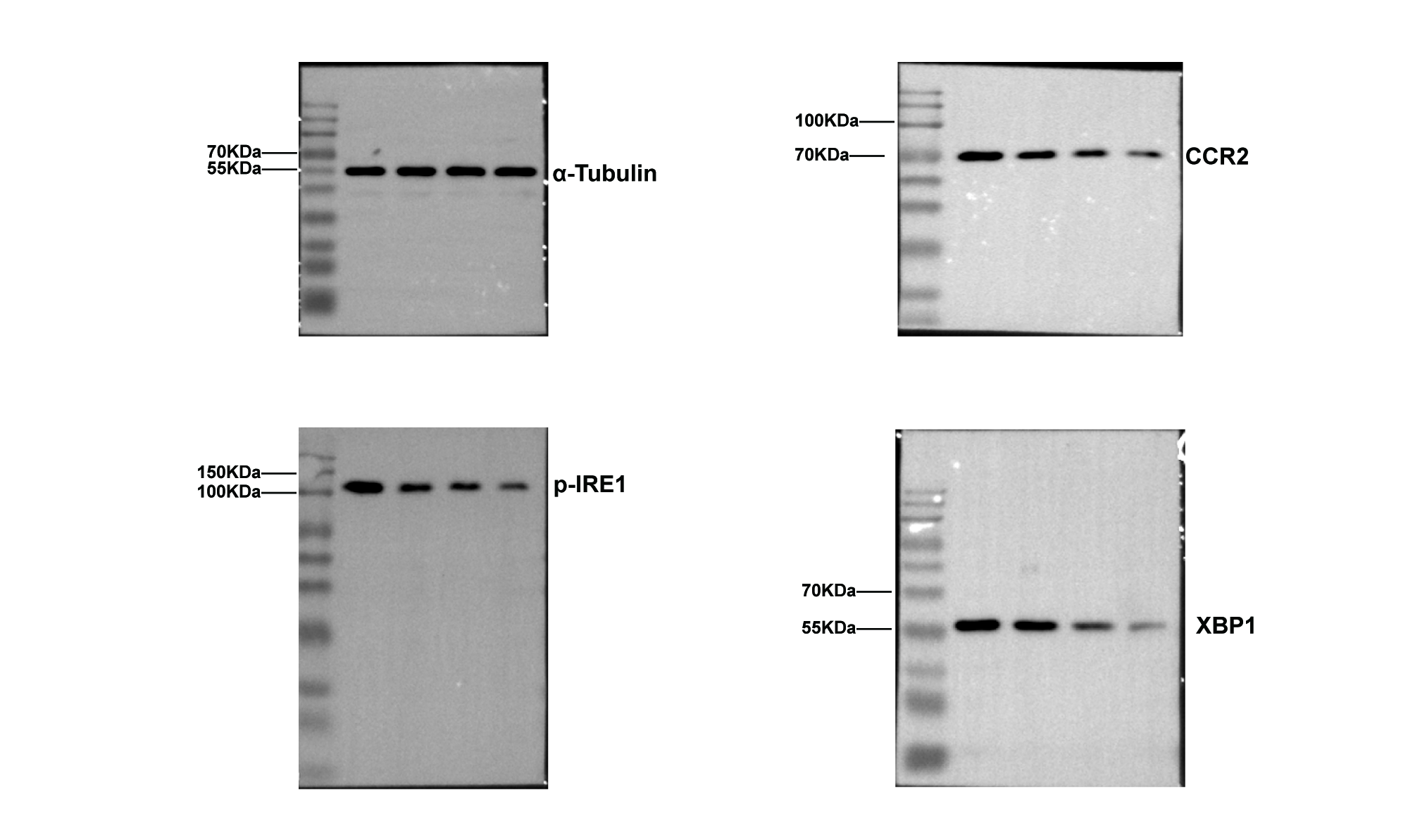


Fig. S8. Western blot full-membrane analysis of NK cell CCR2, p-IRE1, and XBP1 in the four groups: non-hydrogel group, GP, GPHCF, and GPHCFD hydrogel groups.


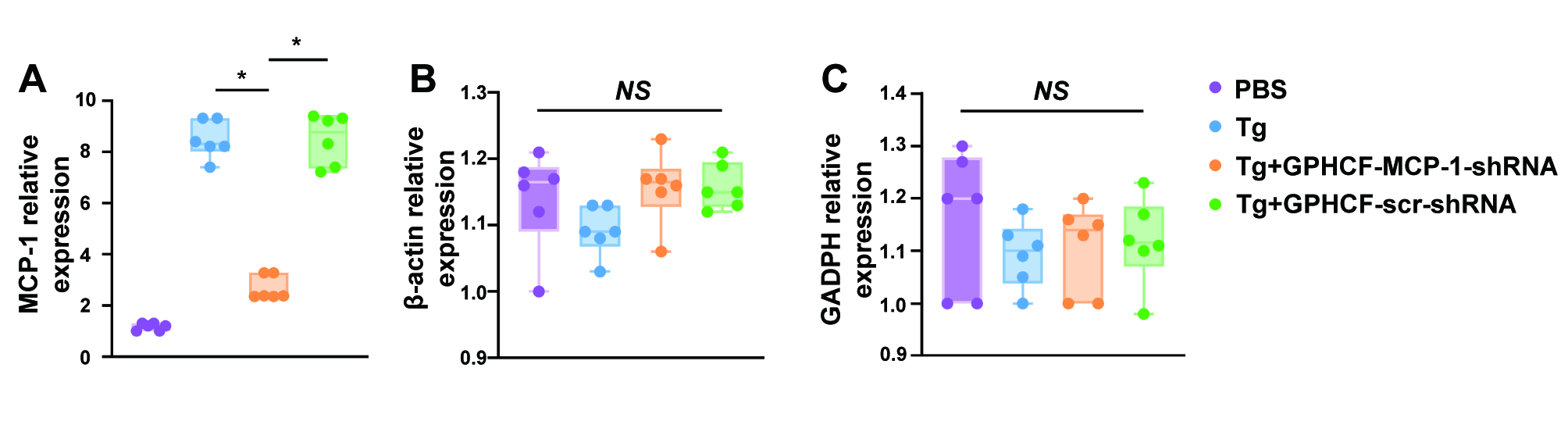


Fig. S9. RT-qPCR detection of off-target effects of MCP-1-shRNA in the GPHCFD Hydrogel.

1. The samples were divided into four groups: PBS, the Tg-treated group, the GPHCF-MCP-1-shRNA (GPHCFD) hydrogel-treated group after Tg-induced excessive ERS, and the GPHCF-scr-shRNA group after Tg-induced excessive ERS. RT-qPCR detected the relative expression levels of MCP-1 in fibroblasts of every group. **B.C.** RT-qPCR detected the relative expression levels of non-target genes β-actin and GAPDH in fibroblasts of every group. n=6 per team. *NS*: non-significant, **P*<0.05. The data is displayed as mean±SD.


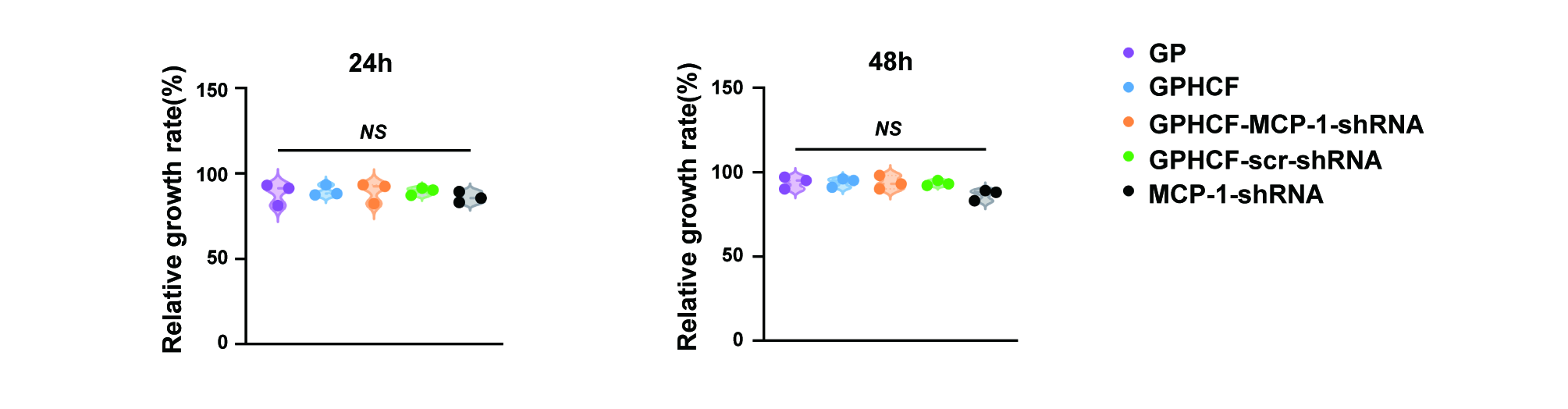


Fig. S10. MTT assay for assessing the cytotoxicity of GPHCFD hydrogel.

The MTT assay determined the relative proliferation rates of fibroblasts treated with the GP, GPHCF, GPHCF-MCP-1-shRNA (GPHCFD), GPHCF-scr-shRNA hydrogel extraction solution, and MCP-1-shRNA solution. n=6 per team. *NS*: non-significant, The data is displayed as mean±SD.


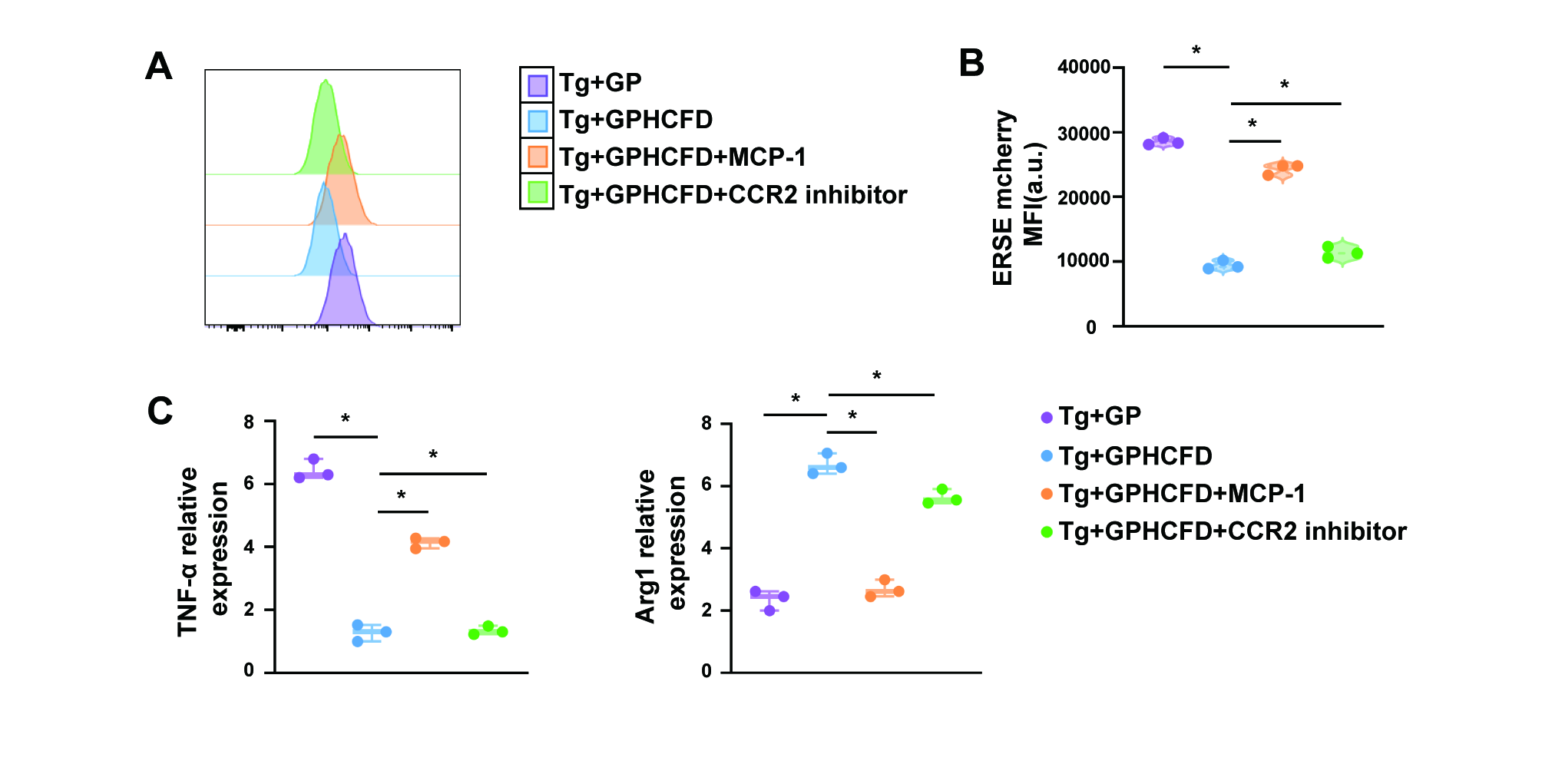


Fig. S11. Supplementing MCP-1 protein attenuated the inhibitory effect of GPHCFD hydrogel on ERS in macrophages and restored its ability to promote the conversion of macrophages from M1 to M2 type.

**A.B.** Co-culture fibroblasts and macrophages. Divide into 4 groups: PBS; Tg-treated group; combined treatment group with Tg, GPHCFD, and MCP-1; combined treatment group with the Tg, GPHCFD, and CCR2 inhibitor. Flow cytometry was used to detect the average fluorescence intensity of ERSE mCherry (ERS reporter mCherry) in macrophages from each group, followed by statistical analysis. **C.** RT-qPCR was used to detect the expression levels of TNF-α and Arg1 in macrophages.n=6 per team. **P*<0.05. The data is displayed as mean±SD.

| **Table S1.** Primer pairs used in the RT-qPCR | |
| --- | --- |
| Genes | Primer sequences |
| GAPDH-F | TCAGCAATGCCTCCTGCAC |
| GAPDH-R | TCTGGGTGGCAGTGATGGC |
| IL-1β-F | CGCCACACCAACTACTCCTT |
| IL-1β-R | TCAGGACCTCAGGGTATGGG |
| TNFα-F | CTGAACTTCGGGGTGATCGG |
| TNFα-R | GGCTTGTCACTCGAATTTTGAGA |
| Arg1-F | ATCAACACTCCCCTGACAAC |
| Arg1-R | GCCAATGTACACGATGTCTTTG |
| iNOS-F | ACATCATGGCACAGGTCAC |
| iNOS-R | TGAGGAAACTGTAAGTCGCTG |
| Β-actin-F | CATGTACGTTGCTATCCAGGC |
| Β-actin-R | CTCCTTAATGTCACGCACGAT |
